# The GSK3/SHAGGY‐like OsGSK3 phosphorylates and inhibits phase separation of OsFCA at Ser‐43 and Ser‐45 to regulate brassinosteroid signaling and rice architecture

**DOI:** 10.1111/nph.71320

**Published:** 2026-06-04

**Authors:** Jiaqi Zhang, Fan Wang, Sijia Zhang, Qian Yu, Qimiao Dong, Jianbo Li, Xianglei Wei, Huaying Du, Ye Shen, Rong Mu, Yanxiao Jia, Jinping Cheng, Hongsheng Zhang, Ji Huang, Xiuying Gao

**Affiliations:** ^1^ State Key Laboratory of Crop Genetics & Germplasm Enhancement and Utilization, College of Agriculture Nanjing Agricultural University Nanjing 211800 China; ^2^ Guangdong Laboratory for Lingnan Modern Agriculture, and the State Key Laboratory for Conservation and Utilization of Subtropical Agro‐Bioresources South China Agricultural University Guangzhou 510642 China; ^3^ Key Laboratory for Enhancing Resource Use Efficiency of Crops in South China, Ministry of Agriculture and Rural Affairs South China Agricultural University Guangzhou 510642 China; ^4^ Jiangsu Provincial Engineering Research Center of Seed Industry Science and Technology Nanjing 210095 China

**Keywords:** brassinosteroid, grain length, heading date, *Oryza sativa* L., phosphoproteomic analysis

## Abstract

- Brassinosteroid (BR) signaling plays a critical role in rice (*Oryza sativa* L.) grain development. GLYCOGEN SYNTHASE KINASE 3 (OsGSK3), a negative regulator of BR signaling, suppresses the transcriptional activity of OsBZR1 through phosphorylation.
- In this study, we employed a phosphoproteomic approach to construct an OsGSK3‐mediated regulatory network. Within this network, we identified FLOWERING CONTROL LOCUS A (OsFCA) as a positive regulator of BR signaling and grain length, with *m*‐*Osfca* mutants exhibiting significantly lower sensitivity to brassinolide treatment. Importantly, OsGSK3 interacts with and phosphorylates OsFCA on serine residues S43 and S45, and then forms condensates via liquid–liquid phase separation. Phosphorylated OsFCA promotes the translocation of the OsGSK3‐OsFCA complex into the cytoplasm. Within the cytoplasm, OsGSK3 and OsFCA no longer exist in a condensate state. This mechanism provides precise regulation of grain length in rice.
- Notably, *Osfca* mutants are late‐flowering, with OsFCA promoting heading under long‐day conditions by repressing the expression of *Grain number, plant height and heading date 7* (*Ghd7*) while activating that of *Early heading date 1* (*Ehd1*), *Heading date 3a* (*Hd3a*), and *RICE FLOWERING LOCUS T 1* (*RFT1*), which is potentially modulated by OsGSK3.
- This study clarifies the BR signaling transduction network by identifying OsFCA as a positive BR signaling component that regulates grain development and heading date, providing theoretical foundations for molecular breeding design in rice.

## Graphical abstract

A working model for GLYCOGEN SYNTHASE KINASE 3‐mediated phosphorylation of FLOWERING CONTROL LOCUS A in the regulation of brassinosteroid signaling and rice growth and development.

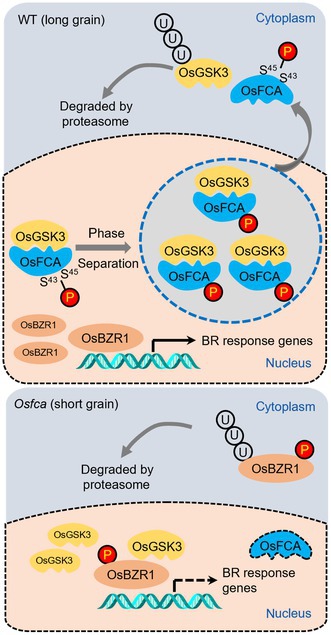

## Introduction

Brassinosteroids (BRs) are natural polyhydroxylated steroids involved in various aspects of plant physiology, including photomorphogenesis, hypocotyl elongation, root growth, formation of organ boundaries, stomatal development, vascular differentiation, male gamete fertility, seed germination, flowering, leaf senescence, and responses to biotic and abiotic stresses (Gendron *et al*., 2012; Hu *et al*., 2021; Manghwar *et al*., 2022). Recent advances in genetics, genomics, and proteomics have significantly enhanced our understanding of BR signal transduction. The genetic modulation of BR levels or signaling components offers innovative strategies for crop improvement.

BR signal perception has been largely elucidated in Arabidopsis (*Arabidopsis thaliana*) and starts with the leucine‐rich repeat (LRR) receptor kinase BR INSENSITIVE 1 (BRI1). BRI1 KINASE INHIBITOR 1 (BKI1), a negative regulator of BR signaling, interacts with the intracellular C‐terminal domain of BRI1 in the absence of brassinolide (BL), blocking the association of the BRI1 kinase domain with its coreceptor BRI1‐ASSOCIATED KINASE 1 (BAK1). The binding of BL to BRI1 induces conformational changes in its kinase domain, leading to the phosphorylation of both BRI1 and BKI1. These phosphorylation events trigger the dissociation of phosphorylated BKI1 from the plasma membrane and the formation of a receptor–coreceptor complex between BRI1 and BAK1 (Li *et al*., 2002; Nam & Li, 2002; Wang & Chory, 2006). The resulting fully activated BRI1 phosphorylates BR SIGNALING KINASEs (BSKs) and CONSTITUTIVE DIFFERENTIAL GROWTH 1 (CDG1), components of BR signaling, propagating the phosphorylation cascade to downstream targets (Tang *et al*., 2008; Kim *et al*., 2011). BSKs and CDG1 activate the protein phosphatase *bri1* SUPPRESSOR 1 (BSU1), which dephosphorylates and inactivates the negative regulator of BR signaling BR INSENSITIVE 2 (BIN2), a Shaggy‐like kinase (Kim *et al*., 2009). BIN2 inactivation promotes the nuclear accumulation of the positive regulators of BR signaling BRASSINOLIDE RESISTANT 1 (BZR1) and *bri1* EMS SUPPRESSOR 1 (BES1). BZR1 and BES1 directly regulate the expression of thousands of downstream genes in response to environmental cues, including light and temperature fluctuations, drought stress, nutrient availability, and pathogen immunity (He *et al*., 2005; Yin *et al*., 2005; Ryu *et al*., 2010; Yu *et al*., 2011).

Rice (*Oryza sativa* L.) shares conserved BR signaling components with Arabidopsis, including the receptor OsBRI1 and the coreceptor OsBAK1, which transmit BR signals to OsBSK3 (Yamamuro *et al*., 2000; Li *et al*., 2009). OsBRI1 phosphorylates OsBSK3, disrupting the autoinhibitory interaction between its tetratricopeptide repeat (TPR) and kinase domains, thereby enhancing the OsBSK3‐BSU1 interaction and activating BR signaling (B. Zhang *et al*., 2016). In contrast with the positive role of Arabidopsis BSU1, its rice ortholog Grain length 3 (qGL3) negatively regulates BR signaling by dephosphorylating OsGSK3 to enhance its stability. The rice OsGSKs protein family consists of nine members, among which OsGSK1‐OsGSK4 redundantly negatively regulate BR signaling, while OsGSK5 is involved in auxin signaling transduction (Yin *et al*., 2025). OsGSK1 is specifically induced by cold, salt stress, and abscisic acid (ABA) treatment (Liu *et al*., 2025). Through either interacting with or phosphorylating distinct substrates, these members are involved in regulating various biological processes such as grain development, leaf angle, lateral root formation, blast resistance, and salt stress response (Tian *et al*., 2021; Hou *et al*., 2022; Meng *et al*., 2024; Liu *et al*., 2025). HDA703, a histone H4 deacetylase, interacts with OsBZR1 to regulate rice BR signaling, growth, and heading date through epigenetic regulation of *Ghd7* (Wang *et al*., 2020).

Beyond OsBZR1, OsGSK2 and its homologs phosphorylate rice‐specific transcription factors such as DWARF AND LOW‐TILLERING (DLT), LEAF AND TILLER ANGLE INCREASED CONTROLLER (LIC), REDUCED LEAF ANGLE 1 (RLA1, also reported as SMALL ORGAN SIZE 1 (SMOS1)), and OVATE FAMILY PROTEINs to modulate their functions (Zhang *et al*., 2012; Hirano *et al*., 2017; Qiao *et al*., 2017; Tong & Chu, 2018; Gruszka, 2020). DLT, a positive regulator of BR signaling belonging to the GAI‐RGA‐SCR (GRAS) family of transcription factors, accumulates in its active dephosphorylated form when the BR signaling cascade inhibits its OsGSK2‐mediated phosphorylation. However, BR also induces the OsBZR1‐mediated repression of *DLT* transcription (Tong *et al*., 2009, 2012). Contrary to the regulation of *DLT* expression by OsBZR1, DLT promotes *OsBZR1* transcription and synergizes with OsBZR1 in a negative feedback loop to regulate the transcription of BR biosynthesis genes. DLT also interacts with ORYZA SATIVA HOMEOBOX 15 (OSH15) to activate *OsBRI1* transcription, highlighting its pleiotropic role in BR homeostasis (Tong *et al*., 2009; Niu *et al*., 2022).

The transition from vegetative to reproductive growth is a critical developmental event, with diversity in flowering time conferring adaptive advantages in natural populations (Blackman, 2017; Gaudinier & Blackman, 2020). In agriculture, flowering time influences crop yield and quality, with the optimal timing of flowering enhancing harvest efficiency and crop marketability (Jung & Müller, 2009). Biomolecular condensates are membrane‐less cellular compartments formed through liquid–liquid phase separation (LLPS), which concentrate biomolecules to facilitate multiple processes, including RNA metabolism and signal transduction (Brangwynne *et al*., 2009; Banani *et al*., 2017). RNA‐binding proteins (RBPs) accompany RNA molecules from synthesis to degradation, modulating each step of their metabolism and impacting a broad range of eukaryotic cellular and developmental processes (Fan *et al*., 2024). Double‐stranded RBP DROUGHT RESISTANCE GENE 9 (DRG9) forms stress granules via LLPS and recruits the mRNA of the key ABA synthesis gene *OsNCED4* to protect it from degradation and thereby enhance rice drought tolerance (Wang *et al*., 2024).

Interaction with the RBP EARLY HEADING DATE 6 (EHD6) triggers part of the m^6^A reader YT521‐B homology 07 (YTH07) migration from the cytoplasm to nonmembranous cytoplasm ribonucleoprotein via phase separation condensation, thus promoting rice flowering (Cui *et al*., 2024). In *A. thaliana*, both the RBP FCA and its homolog SISTER OF FCA (SSF) regulate flowering time via LLPS (Fang *et al*., 2019; Wang *et al*., 2023). FCA promotes flowering by suppressing expression of the flowering repressor *FLOWERING LOCUS C* (*FLC*) (Simpson *et al*., 2003). The coiled‐coil protein FLOWERING LOCUS C EXPRESSOR LIKE 2 (FLL2) plays a crucial role in facilitating FCA phase separation, which is critical for regulating the alternative polyadenylation of antisense *FLC* transcripts (Fang *et al*., 2019). SSF delays flowering by promoting *FLC* transcription, and its interacting protein DECAPPING 5 (DCP5) also undergoes phase separation and can enhance the phase separation of SSF (Wang *et al*., 2023). Moreover, phosphorylation modification affects the phase separation state. Phosphorylation at Ser403 drives polyubiquitin chain‐induced phase separation, the scaffold protein p62 body assembly, and subsequent autophagic degradation (Sun *et al*., 2018).

In this study, we employed a phosphoproteomic analysis of the rice *japonica* cultivar Dongjin (DJ) and the T‐DNA insertion mutant *m‐Osgsk3* of *OsGSK3* to identify phosphorylation substrates of OsGSK3. Among these proteins, we focused on FLOWERING CONTROL LOCUS A (OsFCA), a regulator of heading date. In Arabidopsis, FCA interacts with FY to suppress *FLOWERING LOCUS C* (*FLC*) transcription, promoting flowering via the autonomous pathway (Xu *et al*., 2021; Qi *et al*., 2022). Heterologous expression of *OsFCA* in Arabidopsis was previously shown to partially rescue the late‐flowering phenotype of *fca* mutants while failing to suppress *FLC* transcription, indicating a limited functional conservation between FCA and OsFCA (Lee *et al*., 2005). In the *japonica* cultivar Zhonghua11 (ZH11), constitutive *OsFCA* expression delays heading, diminishes plant height, increases grain weight, and induces awn formation (Attia *et al*., 2005). In this study, we demonstrate that loss of OsFCA function results in shorter grains and delayed flowering, providing insights into how the OsGSK3‐OsFCA interaction regulates grain length and heading date.

## Materials and Methods

### Plant materials and growth conditions

The rice (*Oryza sativa* L. subsp. *japonica*) cultivars DJ and ZH11 were used as background for genetic transformation. The *m*‐*Osfca* T‐DNA insertion mutant (PFG_3A‐02240.R) and *m‐Osgsk3* in the DJ background was obtained from the Korean rice mutant library (https://signal.salk.edu). The gene‐edited materials were obtained in the ZH11 background by genetic transformation in the laboratory reference to the method published by Xing *et al*. (2014). Using the website (http://www.genome.arizona.edu/crispr/CRISPRsearch.html) to screen the gene editing sites of target genes, two target sites can be selected at the same time, and the Protospacer Adjacent Motif must be "NGG" (N represents any base). Homology alignment was performed to determine the target specificity. Rice plants were cultivated in the field under natural May to October in Nanjing, China. The validation primers for T‐DNA insertion mutants and the gene‐edited materials are listed in Supporting Information Table S1.

### Preparation of samples for phosphoproteomic analysis

The samples from 4 to 6‐wk‐old leaves from DJ, DJ treated with 10^−7^ M BL, and *m‐Osgsk3* were ground to a powder in liquid nitrogen; the powder was mixed with an appropriate volume of guanidine hydrochloride lysis buffer. Homogenization was performed using a tissue homogenizer, and the lysate was transferred to an Eppendorf tube. Samples were then heated in a boiling water bath for 3 min, followed by sonication for 2 min. After centrifugation at 16 000 ***g*** for 20 min at 4°C, the supernatant was collected, and the protein concentration was determined using a bicinchoninic acid (BCA) assay. An appropriate amount of protein from each sample was subjected to tryptic digestion using the Filter‐Aided Sample Preparation method. Briefly, dithiothreitol (DTT) was added to a final concentration of 100 mM, followed by heating in a boiling water bath for 5 min and cooling to room temperature. Then, 200 μl of Urea (UA) buffer (8 M urea, 150 mM Tris–HCl, pH 8.0) was added and the mixture was transferred to a 10‐kDa ultrafiltration tube and centrifuged at 12 000 ***g*** for 15 min at 4°C. The sample in the filter of the filtration tube was washed once with another 200 μl of UA buffer and centrifuged again under the same conditions. Next, 100 μl of indole‐3‐acetic acid solution (50 mM iodoacetamide in UA buffer) was added to the filter, mixed at 600 rpm for 1 min, and incubated in the dark at room temperature for 30 min. After centrifugation at 12 000 ***g*** for 10 min, the filter was washed twice with 100 μl of UA buffer and twice with 100 μl of 50 mM NH_4_HCO_3_, each time followed by centrifugation at 14 000 ***g*** for 10 min at 4°C. Then, 40 μl of trypsin solution (6 μg trypsin in 40 μl 50 mM NH_4_HCO_3_) was added to each sample, followed by mixing at 600 rpm for 1 min and incubation at 37°C for 16–18 h. After digestion, the peptides were collected by centrifugation at 12 000 ***g*** for 10 min into a new tube. The resulting filtrate was acidified with 0.1% (v/v) trifluoroacetic acid and desalted using a C18 cartridge, followed by vacuum drying. Dried peptides were reconstituted in 0.1% (v/v) formic acid, and peptide concentration was measured using a NanoDrop spectrophotometer. The reconstituted peptides were again dried and subjected to phosphopeptide enrichment using an Fe‐NTA Phosphopeptide Enrichment Kit (A32992; Thermo Fisher Scientific, Waltham, MA, USA) following the manufacturer's instructions. The eluted phosphopeptides were vacuum‐concentrated, reconstituted in 10 μl of 0.1% (v/v) formic acid, and used for LC‐MS/MS analysis.

### Data‐independent acquisition (DIA) mass spectrometry

An appropriate number of peptides from each sample was subjected to chromatographic separation using a Vanquish Neo UHPLC system (Thermo Fisher Scientific). The mobile phases consisted of Buffer A (0.1% [v/v] formic acid in water) and Buffer B (0.1% [v/v] formic acid in 80% [v/v] acetonitrile), and the analytical column was equilibrated with 96% (v/v) Buffer A before sample injection. Peptides were first loaded onto a trap column (PepMap Neo, 5 μm C18, 300 μm × 5 mm; Thermo Fisher Scientific), then separated on a μPAC Neo High Throughput analytical column (Thermo Fisher Scientific) under the following gradient conditions (all in v/v): 0–0.1 min, Buffer B increasing from 4% to 6%; 0.1–1.1 min, 6% to 12%; 1.1–4.3 min, 12% to 22.5%; 4.3–6.1 min, 22.5% to 45%; and 6.1–8.0 min, Buffer B maintained at 99%. After separation, peptides were analyzed using the data‐independent acquisition (DIA) mode on an Orbitrap Astral mass spectrometer (Thermo Fisher Scientific). The total analysis time was 8 min. Electrospray ionization was conducted at 2.2 kV in positive ion mode. The MS1 scan range was *m/z* 380–980, with a resolution of 240 000, an automatic gain control (AGC) target of 500%, and a maximum injection time (IT) of 3 msec. MS2 scans were acquired at a resolution of 80 000 with an AGC target of 500%, a maximum IT of 3 ms, a radio frequency (RF) lens setting of 40%, and higher energy collisional dissociation (HCD) with a normalized collision energy of 25%. The isolation window was set to 2 Th, and the cycle time was 0.6 s.

### Direct DIA analysis

All raw mass spectrometry data were processed and analyzed using spectronaut software (v.18; Biognosys AG, Schlieren, Switzerland). The data from all samples were combined and searched against a reference spectrum library generated during the analysis. The protein database used was uniprot kb_*Oryza sativa* subsp. *japonica* (Rice) [39947]‐149 130‐20 231 102.fasta, obtained from the UniProt database (https://www.uniprot.org/39947).

### Protein identification and quantification results

For analysis of DIA‐based protein identification and quantification data, each sample was independently prepared and subjected to DIA analysis after protein digestion. The resulting raw DIA mass spectrometry files were imported into the spectronaut software for analysis, using a *Q*‐value ≤ 0.01 as the filtering threshold.

### Data analysis and visualization

Venn diagrams were generated using the online tool Venny 2.1 (https://bioinfogp.cnb.csic.es/tools/venny/) to define the extent of overlap of differentially phosphorylated proteins between groups. Differentially phosphorylated sites were defined as those with a fold change ≥ 1.5 and *P* value < 0.05.

Principal component analysis (PCA) was performed using Z‐score‐normalized abundance data of total and phosphorylated proteins using the PCA module in scikit‐learn (Python). The first two principal components were plotted to visualize sample clustering. PCA plots were generated with Seaborn and Matplotlib.

Volcano plots were generated with the ggplot2 package in R. Log_2_‐transformed fold changes and ‐log_10_‐transformed *P* values were calculated for each protein or phosphorylation site. Heatmaps were generated using the pheatmap package in R based on normalized protein abundance data.

The protein–protein interaction (PPI) network was visualized using the STRING database and Cytoscape software. Functional classifications related to OsGSK3‐regulated proteins were retrieved from the STRING database.

### Yeast two‐hybrid (Y2H) assays

The full‐length coding sequence (CDS) of *OsFCA*, *OsSPL15*, and *OsCesA4* was cloned into the pGADT7 vector to obtain the prey construct AD‐OsFCA, AD‐OsSPL15, and AD‐OsCesA4. The full‐length CDS of *OsGSK3* was cloned into the pGBKT7 vector to generate the bait construct BD‐OsGSK3. The appropriate combinations of plasmids were co‐introduced into the yeast (*Saccharomyces cerevisiae*) strain AH109 according to the manufacturer's instructions (Clontech, Carlsbad, CA, USA). Positive transformants were selected for growth on synthetic defined (SD) medium lacking tryptophan and leucine (SD/‐Trp‐Leu) at 30°C for 3 d. PPI were tested by spotting serially diluted cultures of each positive transformant onto SD medium lacking tryptophan, leucine, histidine, and adenine (SD/‐Trp‐Leu‐His‐Ade) and containing 4 mg ml^−1^ 5‐bromo‐4‐chloro‐3‐indolyl‐α‐D‐galactoside (X‐α‐gal) (SL0940; Coolaber, Beijing, China). The PCR primers used for the cloning steps in the yeast two‐hybrid (Y2H) assay are listed in Table S1.

### Bimolecular fluorescence complementation (BiFC) assays

For bimolecular fluorescence complementation (BiFC) assays, the full‐length CDS of *OsFCA*, *OsGSK3*, and *SDG725* were cloned into the p2YC and p2YN vectors, respectively, resulting in the constructs OsFCA‐YC, OsGSK3‐YN, SDG725‐YC, OsFCA‐YN, and OsGSK3‐YC. The resulting plasmids and empty vectors were individually introduced into Agrobacterium (*Agrobacterium tumefaciens*) strain EHA105. Positive Agrobacterium colonies were grown in LB medium with the appropriate antibiotics overnight, resuspended in infiltration buffer (10 mM MgCl_2_; 10 mM MES, pH 5.7; 80 μM Acetosyringone), and the appropriate combinations of two cell suspensions were mixed in a 1 : 1 ratio (v/v) before being infiltrated into the young leaves of *Nicotiana benthamiana* plants. Yellow fluorescent protein (YFP) fluorescence was observed under a Zeiss LSM780 confocal microscope (514 nm Argon, collection bandwidth: 525–620 nm, gains: PMT 750 V, Digital Gain 1.0) after growth for 36–48 h in darkness. The PCR primers used for the cloning steps in the BiFC assay are listed in Table S1.

### Glutathione S‐transferase (GST) pull‐down assay

To test the interaction between OsFCA and OsGSK3 *in vitro*, the full‐length CDS of *OsFCA* was cloned into the pMAL‐C2X vector; the resulting construct was introduced into the *Escherichia coli* strain BL21 (DE3) to produce the MBP‐OsFCA fusion protein. The plasmid GST‐OsGSK3 (original vector backbone pGEX‐2T vector) was described previously (Gao *et al*., 2019). The production of the fusion proteins MBP‐OsFCA and GST‐OsGSK3 was induced by the addition of 0.5 mM isopropyl‐b‐D‐thiogalactopyranoside and incubation at 18°C for 12 h. For glutathione S‐transferase (GST) pull‐down assays, bacterial lysates containing GST‐OsGSK3 or GST were mixed with lysates containing MBP‐OsFCA. Subsequently, GST Bind Resin (Novagen, Darmstadt, Germany) was added to the mixtures and incubated at 4°C for 4 h. Beads were washed three times with PBS buffer and then boiled in 1 × SDS loading buffer for 10 min. Finally, the boiled samples were separated by electrophoresis on 10% (w/v) SDS‐PAGE gels. An anti‐MBP antibody (Cell Signaling Technology, Danvers, MA, USA) and an anti‐GST antibody (Cell Signaling Technology) were used to detect the proteins by immunoblot analysis. The PCR primers used for the cloning steps in the GST pull‐down assay are listed in Table S1.

### RNA Extraction and Real‐time quantitative PCR (RT‐qPCR)

Total RNA was isolated using the FastPure Universal Plant Total RNA Isolation Kit (RC411‐01; Vazyme, Nanjing, China). Subsequently, cDNA synthesis was performed with HiScript III 1st Strand cDNA Synthesis Kit (R312‐01; Vazyme) according to the manufacturer's instructions. Real‐time quantitative PCR (RT‐qPCR) was conducted in 96‐well plates on a Light Cycler 480 real‐time system (Roche, Basel, Switzerland) using ChamQ SYBR qPCR Master Mix (Q331‐02; Vazyme). Rice *OsActin* was used as an internal control. The relative gene expression levels were calculated using the 2^−ΔΔCT^ method. Experiments were performed with three biological replicates. The primers used for RT‐qPCR analysis are listed in Table S1.

### *In vitro* phosphorylation assays

One microgram of purified MBP‐OsFCA and 0.5 μg of GST‐OsGSK3 were added to the phosphorylation buffer (20 mM HEPES, 150 mM NaCl, 10 mM MgCl_2_, 1 mM Adenosine 5′‐(trihydrogen diphosphate) (ATPγS) (ab138911; Abcam, Cambridge, Britain)). 50 μM Bikinin was used to inhibit OsGSK3 kinase activity. Mix thoroughly by pipetting and incubate at room temperature for 30 min. 2.5 mM p‐nitrobenzenesulfonamide (ab138910; Abcam) was added to the system to alkylate the substrate, and the mixture was incubated at room temperature for 60 min. Add an appropriate volume of 5 × SDS loading buffer to the system and denature at 95°C for 5 min. Perform western blotting using an anti‐Thiophosphate ester antibody (ab92570; Abcam) to detect the phosphorylation status of OsGSK3 and OsFCA.

### Brassinolide (BL)‐induced expression analysis

For BL‐induced expression analysis, full‐grained DJ seeds were soaked in water for 2–3 d, germinated, and sown in 96‐well PCR plates with the bottom of each well cut. The seedlings were grown hydroponically in a climate‐controlled glasshouse, under a 14 h : 10 h, 28°C : 24°C, light : dark cycle for 1 wk with Yoshida nutrient solution (Yoshida *et al*., 1971). 24‐Epibrassinolide (24‐eBL) powder was dissolved in Dimethyl sulfoxide (DMSO) to a stock solution of 50 μM, which was added to the nutrient solution to achieve a final 24‐eBL concentration of 1 μM, with the control group receiving an equivalent volume of DMSO. The aboveground tissues of rice seedlings were sampled immediately before 24‐eBL addition and at 0.5, 1, 2, 3, and 6 h following 24‐eBL addition, and total RNA was extracted and analyzed by real‐time quantitative PCR (RT‐qPCR).

### Lamina inclination assays

For lamina inclination assays in response to BL treatment, full‐grained rice seeds were soaked in water for 2–3 d, germinated, and sown on a substrate of equal parts nutrient soil and vermiculite. After one week of growth in a climate‐controlled glasshouse under a 14 h : 10 h, 28°C : 24°C, light : dark cycle, segments consisting of the second leaf blade, lamina joint, and 2 cm of leaf sheath was cut off and submerged in a 1 μM 24‐eBL solution. The angles of lamina joint bending were measured after incubation for 72 h in darkness.

### Analysis of diurnal expression pattern of heading date‐related genes

Fifty seeds each of DJ, *m*‐*Osfca*, ZH11, and *Osfca‐3* were soaked in water for 2–3 d, and the seedlings were sown onto a substrate of equal parts nutrient soil and vermiculite, and placed in a climate‐controlled glasshouse under a 14 h : 10 h, 28°C : 24°C, light : dark photoperiod cycle for growth for 55 d. Rice flag leaves were sampled at 4‐h intervals, beginning at ZT0 (lights on). Total RNA was extracted for RT‐qPCR to detect diurnal rhythms in the transcript levels of heading date‐related genes.

### Cell‐free protein degradation assay

Total proteins were extracted from 2‐wk‐old wild‐type (WT), *Osgsk3*, and *OE‐OsGSK3* seedlings using a lysis buffer (25 mM Tris–HCl, pH 7.5; 10 mM MgCl_2_; 10 mM NaCl; 5 mM DTT; 1 mM PMSF). Equal amounts of purified MBP‐OsFCA protein were incubated with protein extracts from wild‐type, *Osgsk3*, and *OE‐OsGSK3* plants supplemented with 10 mM ATP at 28°C for different time gradients. MG132 was added to inhibit the ubiquitination‐dependent protein degradation. The reaction was terminated by adding an appropriate volume of 5 × SDS loading buffer, followed by denaturation at 95°C for 5 min. Then, the proteins were separated by SDS‐PAGE. The stability of MBP‐OsFCA protein in the samples was assessed via western blot analysis using anti‐MBP antibody (Cell Signaling Technology), with Rubisco serving as the internal control.

### Fluorescence recovery after photobleaching (FRAP)

FRAP assays were performed on tobacco epidermal cells co‐expressing OsFCA‐GFP, OsFCA^AA^‐GFP, or OsFCA^DD^‐GFP together with OsGSK3‐YC. A selected region of OsFCA‐GFP droplets was photobleached with a 488‐nm laser pulse (10 iterations, 100% intensity), and the droplets' dynamics along with FRAP images were captured using Zeiss LSM900 confocal microscope. The fluorescence recovery was quantified by normalizing the intensity at each time point to the value before photobleaching.

### RNA‐Seq analysis

Young panicles of ZH11, *Osfca‐2*, and *Osgsk3 Osfca‐2* were sampled for RNA‐seq analysis, with three independent biological replicates per genotype. The extraction and examination of total RNA, library construction, and high‐throughput sequencing were conducted by Wuhan SeqHealth Tech Co., Ltd (Wuhan, China). The enriched libraries were quantified and sequenced on a DNBSEQ‐T7 platform (MGI) using the PE150 sequencing mode to generate paired‐end reads. The clean reads were aligned with reference sequences of rice in IRGSP‐1.0 (http://rapdb.dna.affrc.go.jp/download/irgsp1.html). Differentially expressed genes (DEGs) were defined by absolute log_2_(Fold Change) > 1 and *P* value < 0.05.

## Results

### In‐depth proteomic and phosphoproteomic profiling of the BR signaling pathway

**Fig. 1:**
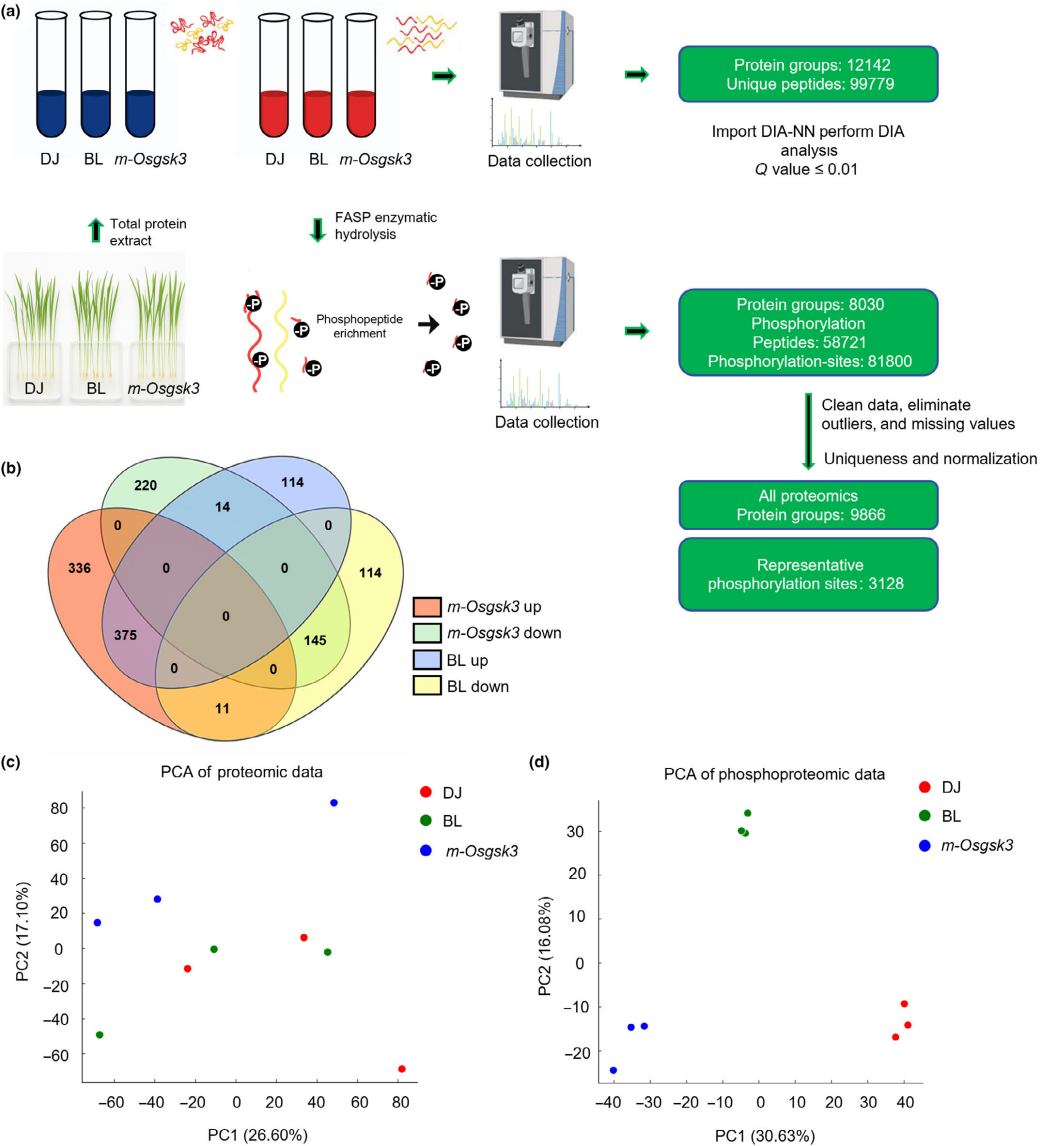
Phosphoproteomic analysis of brassinosteroid signaling in rice (*Oryza sativa* L.) using wild‐type (WT), brassinolide (BL)‐treated WT, and *m‐Osgsk3* mutant plants. (a) Experimental workflow and analysis for data‐independent acquisition‐based quantitative analysis of protein phosphorylation modifications. (b) Venn diagrams showing the extent of overlap in proteins with greater or lower phosphorylation abundance between the BL‐treated (DJ_BL) vs DJ and *m‐Osgsk3* vs DJ comparisons. (c, d) Principal component analysis of the proteome (c) and phosphoproteome (d) data.

To comprehensively investigate the potential mechanisms of the BR signaling pathway in rice, we performed proteomic and phosphoproteomic quantitative analyses with WT plants, WT plants (DJ cultivar) treated with BL, and the T‐DNA insertion mutant *m‐Osgsk3* of *OsGSK3* (Fig. 1a). We detected and identified phosphorylated proteins in these samples using iTRAQ‐based liquid chromatography–tandem mass spectrometry (LC‐MS/MS), following phosphopeptide enrichment with an Fe‐NTA Phosphopeptide Enrichment Kit. We identified a total of 12 142 proteins (Table S2) and 99 779 unique peptides (Table S3). In the phosphoproteome dataset, we detected 58 721 phosphopeptides (Table S4) and 81 800 phosphorylation sites (Table S5). In subsequent analyses, we retained proteins showing no differences in their protein levels but changes in their phosphorylation status for in‐depth analysis. To minimize the influence of total protein abundance on phosphorylation quantification, the intensity of each phosphosite was further normalized by its corresponding total protein abundance, followed by a log_2_ transformation of the resulting normalized phosphorylation status values. As a result, we obtained 9866 quantifiable protein entries (Table S6) and 3128 representative phosphorylation sites (Table S7).

We determined the number of proteins with more or fewer detected phosphorylation sites in the comparisons between the BL‐treated WT samples and the WT, and between the *m‐Osgsk3* mutant and the WT, using Venn diagrams for visualization (Fig. 1b). Compared with the control DJ group of samples treated with DMSO only, there were 503 upregulated phosphorylated proteins and 270 downregulated phosphorylated proteins in the BL‐treated DJ samples. By contrast, the *m‐Osgsk3* group of samples was characterized by 722 upregulated phosphorylated proteins and 379 downregulated phosphorylated proteins relative to DJ. We performed a PCA on the proteomic and phosphoproteomic datasets (Fig. 1c,d). Samples from the three groups (DJ, DJ_BL, and *m‐Osgsk3*) were clearly separated based on their global protein abundance profiles, indicating distinct proteomic patterns among the groups.

Based on previous reports, we noticed that the phenotype and gene expression patterns in the *m‐Osgsk3* mutant resembled those observed in WT plants treated with BL, indicative of enhanced BR signaling. To identify proteins associated with OsGSK3 and BR signaling, we selected proteins showing the same regulatory trend in the DJ BL‐treated and *m‐Osgsk3* mutant datasets. We thus identified 229 proteins with consistent regulation for analysis (Table S8) and performed a hierarchical clustering of these proteins and visualized their abundance patterns as a heatmap (Fig. S1).

### Construction of an OsGSK3‐dependent network

**Fig. 2:**
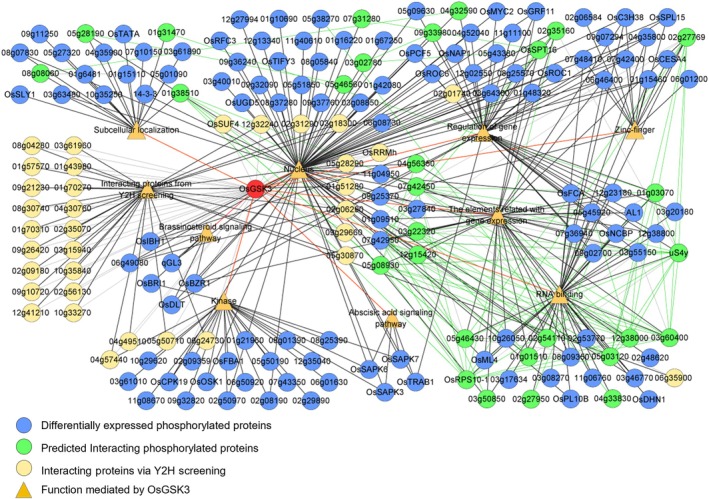
The GLYCOGEN SYNTHASE KINASE 3‐dependent network. Green lines connect proteins predicted to interact based on experiments active interaction source in the STRING database. Light gray lines connect proteins predicted to interact based on text mining, databases, co‐expression, neighborhood, gene fusion, and co‐occurrence active interaction sources in the STRING database.

We constructed a PPI network with OsGSK3 as the central node to reveal its regulatory roles in multiple functional modules (Fig. 2). This PPI network comprised 178 proteins (Table S9), among which 29 PPI were supported by prior experimental evidence. The network demonstrated that OsGSK3 (1) modulates BR signaling via direct targets such as OsBZR1 and qGL3, and (2) influences broader pathways including regulation of gene expression, phosphorylation events, and ABA signaling. These findings underscore the role of OsGSK3 as a multifunctional kinase involved in intricate signaling crosstalk. As illustrated in the network, OsGSK3 is associated with a wide range of biological functions. Notably, the OsGSK3‐interacting proteins OSMOTIC STRESS/ABA‐ACTIVATED PROTEIN KINASE 3 (OsSAPK3), OsSAPK6, OsSAPK7, and TRANSCRIPTION FACTOR RESPONSIBLE FOR ABA REGULATION 1 (OsTRAB1) are all known regulators in the ABA signaling pathway. OsSAPK3, OsSAPK6, and OsSAPK7 are also enriched in functions related to phosphorylation and kinase activity (Fig. 2).

Additionally, we identified proteins such as ALBINO LEAF 1 (AL1), OsFCA, and uS4y as OsGSK3‐regulated components involved in RNA binding, regulation of gene expression, and subcellular localization as putative direct interactors and substrates of OsGSK3 (Fig. 2). AL1 regulates chloroplast development in a dose‐dependent manner, with its abundance gradually rising during germination and the protein primarily accumulating in mature leaves, while also being detected in roots, leaf sheaths, seeds, stems, panicles, and roots (Z. Zhang *et al*., 2016). uS4y, a small ribosomal subunit, interacts with multiple proteins within the network.

The network also revealed that OsGSK3 regulates a series of zinc finger proteins, such as SQUAMOSA PROMOTER BINDING PROTEIN‐LIKE 15 (OsSPL15). Another putative substrate of OsGSK3 was cellulose synthase catalytic subunit genes 4 (OsCesA4); together with OsCesA7 and OsCesA9, these proteins represent three distinct catalytic subunits of cellulose synthase that are not functionally redundant and are likely to form a cellulose synthase complex involved in secondary cell wall formation (Tanaka *et al*., 2003). Recent studies suggest that OsCesA4, OsCesA7, and OsCesA9 may function downstream of Grain Size 3 (GS3), potentially mediating resistance to brown plant hoppers (*Nilaparvata lugens*) (Shen *et al*., 2023).

### OsFCA positively regulates grain length and the BR signaling pathway

To investigate whether OsGSK3 physically interacts with proteins differentially phosphorylated by OsGSK3, we selected three candidate proteins – OsFCA, OsCesA4, and OsSPL15 – for validation. The results demonstrated that OsGSK3 exhibits a strong interaction with OsFCA, and a weak but detectable interaction with OsSPL15 (Fig. S2). Accordingly, we purchased the T‐DNA insertion mutant of *OsFCA*. We confirmed the presence of a T‐DNA insertion in the *OsFCA* locus in this *m‐Osfca* mutant (Fig. S3a–c). Compared with its WT DJ, the *m*‐*Osfca* mutant plants were shorter by 13.2% (Fig. S3d,e) and were observed shorter and rounder grains characteristic of loss of BR signaling or biosynthesis (Fig. S3f–i). The *m*‐*Osfca* mutant was more compact than the WT, prompting us to speculate that OsFCA regulates leaf angle in rice. Indeed, the angle between the second top leaf and the main stem was 16.2° narrower in *m‐Osfca* compared with that in DJ at the grain‐filling stage (Fig. S3j,k). RT‐qPCR analysis showed that the transcript levels of the BR biosynthesis genes *CONSTITUTIVE PHOTOMORPHOGENIC DWARF 1* (*CPD1*), *OsDWARF*, and *DWARF2* (*OsD2*) were higher in *m‐Osfca* plants than in DJ plants, while the transcript levels of *OsDWARF4* were lower in the mutant (Fig. S3l). The results indicate that the loss of OsFCA function affects the expression of BR biosynthesis genes.

### OsGSK3 interacts with and phosphorylates OsFCA

**Fig. 3:**
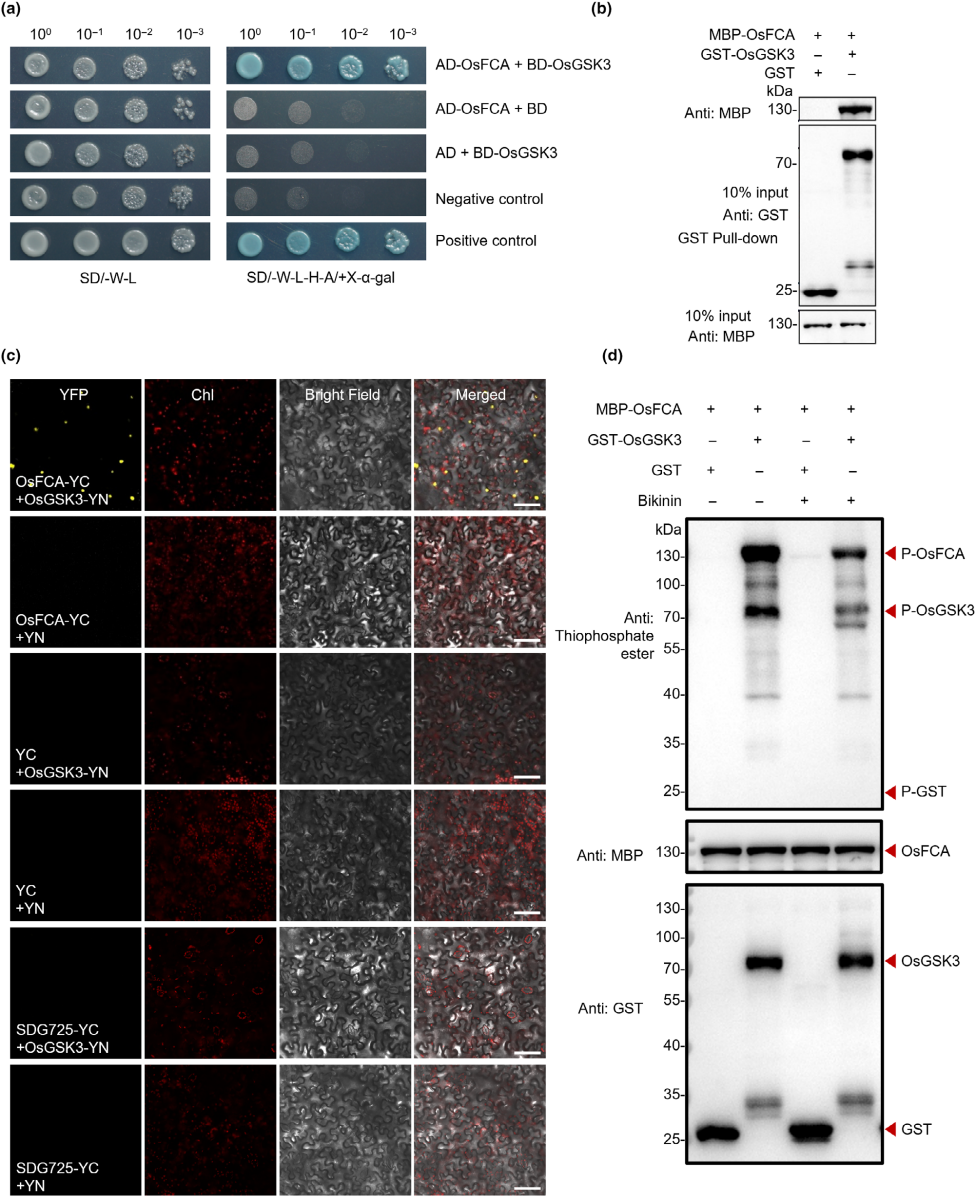
FLOWERING CONTROL LOCUS A (OsFCA) interacts with and is phosphorylated by GLYCOGEN SYNTHASE KINASE 3 (OsGSK3). (a) Yeast two‐hybrid (Y2H) assay showing that OsFCA interacts with OsGSK3 in yeast cells. Yeast cultures carrying the indicated combinations of plasmids were serially diluted with ddH_2_O (10^0^, 10^−1^, 10^−2^, 10^−3^) and spotted onto synthetic defined (SD) medium lacking tryptophan and leucine (SD/−W−L) and SD medium lacking tryptophan, leucine, histidine, and adenine, and containing X‐α‐gal (SD/−W−L−H−A/+X‐α‐gal). (b) Glutathione S‐transferase (GST) pull‐down assay showing that GST‐OsGSK3, but not GST, pulls down MBP‐OsFCA *in vitro*. Immunoblot analysis was performed with anti‐MBP and anti‐GST antibodies. (c) Bimolecular fluorescence complementation assay of the interaction between OsFCA and OsGSK3 in the leaf epidermal cells of *Nicotiana benthamiana* plants. YFP, yellow fluorescent protein; Chlorophyll, Chl autofluorescence; Merged, merged fields. Bars, 100 μm. (d) OsGSK3 phosphorylates OsFCA *in vitro*. 50 μM Bikinin was used to inhibit OsGSK3 kinase activity. The anti‐MBP antibody was used to detect OsFCA; anti‐GST antibody was used to detect OsGSK3 and GST; anti‐thiophosphate ester antibody was used to detect the phosphorylated protein.

OsGSK3 is a negative regulator of BR signaling by phosphorylating OsBZR1 and inhibiting the transmission of BR signals (Gao *et al*., 2019). To further validate the interaction and regulatory relationship between OsGSK3 and OsFCA, we conducted an in‐depth investigation. First, we performed a Y2H assay, which showed that OsFCA can interact with OsGSK3 (Fig. 3a). We turned to a GST pull‐down assay to confirm the interaction of OsFCA and OsGSK3. To this end, we produced maltose‐binding protein (MBP)‐OsFCA and GST‐OsGSK3 in *E. coli* and purified the soluble proteins for GST pull‐downs. Immunoblot assays failed to detect the target band for MBP‐OsFCA after co‐incubation with GST alone; however, we did detect MBP‐OsFCA among the proteins pulled down by GST‐OsGSK3. These results indicate that OsFCA can interact with OsGSK3 *in vitro* (Fig. 3b). Then we verified the interaction between OsFCA and OsGSK3 by BiFC assays, with yellow fluorescence detected only in the nucleus of *N. benthamiana* leaf epidermal cells co‐expressing *OsFCA‐YC* (encoding a fusion of OsFCA and the C‐terminal half of YFP) and *OsGSK3‐YN* (encoding a fusion of OsGSK3 and the N‐terminal half of YFP). Importantly, the co‐expression of *OsFCA‐YC* and *YN* (encoding the N‐terminal half of YFP only), or *OsGSK3‐YN* and *YC* (encoding the C‐terminal half of YFP only) or *OsGSK3‐YN* and *SDG725‐YC* in *N. benthamiana* epidermal cells resulted in no detectable YFP fluorescence, indicating that OsFCA and OsGSK3 interact in the nucleus (Fig. 3c). As OsFCA and OsGSK3 physically interacted *in vitro* and *in vivo*, and we had identified OsFCA as a putative phosphorylation substrate of OsGSK3, we conducted an *in vitro* phosphorylation assay to assess whether OsGSK3 can phosphorylate OsFCA. Indeed, an ATPγS assay revealed the OsGSK3‐dependent phosphorylation of OsFCA (Fig. 3d). The phosphorylation levels of OsGSK3 and OsFCA decreased following the addition of bikinin (Fig. 3d), an OsGSK3 inhibitor, suggesting that OsFCA may be a downstream substrate of OsGSK3.

### The genetic relationship between *OsGSK3* and *OsFCA*

To study the genetic relationship between *OsGSK3* and *OsFCA* in the regulation of grain length, we generated *Osfca*, *Osgsk3*, and *Osgsk3 Osfca* mutants via CRISPR/Cas9‐mediated gene editing of *OsGSK3* or *OsFCA* genes in the *japonica* background ZH11, respectively. To generate *Osfca* materials, the target site of the single‐guide RNA was located within exon 1 of *OsFCA* (Fig. S4a). Sequencing of T0‐generation *Osfca* material at the Target 1 site identified three plants with mutations at this site, namely a 1‐bp insertion (of an A nucleotide; *Osfca‐1*), a 1‐bp insertion (of a T nucleotide; *Osfca‐2*), and a 28‐bp insertion (*Osfca‐3*) (Fig. S4b). We obtained two mutants of *OsGSK3*, one with a 1‐bp insertion (of a G nucleotide; *Osgsk3‐1*) and one with a 4‐bp deletion (of the sequence TATG; *Osgsk3‐2*), both leading to premature termination of OsGSK3 translation (Fig. S5a,b). We also identified two independent *Osgsk3 Osfca* mutant lines, both with a 1‐bp insertion in *OsFCA* (of an A or T nucleotide) and a 1‐bp deletion in *OsGSK3* (of a G nucleotide), leading to premature termination of OsFCA and OsGSK3 translation (Fig. S5c).

**Fig. 4:**
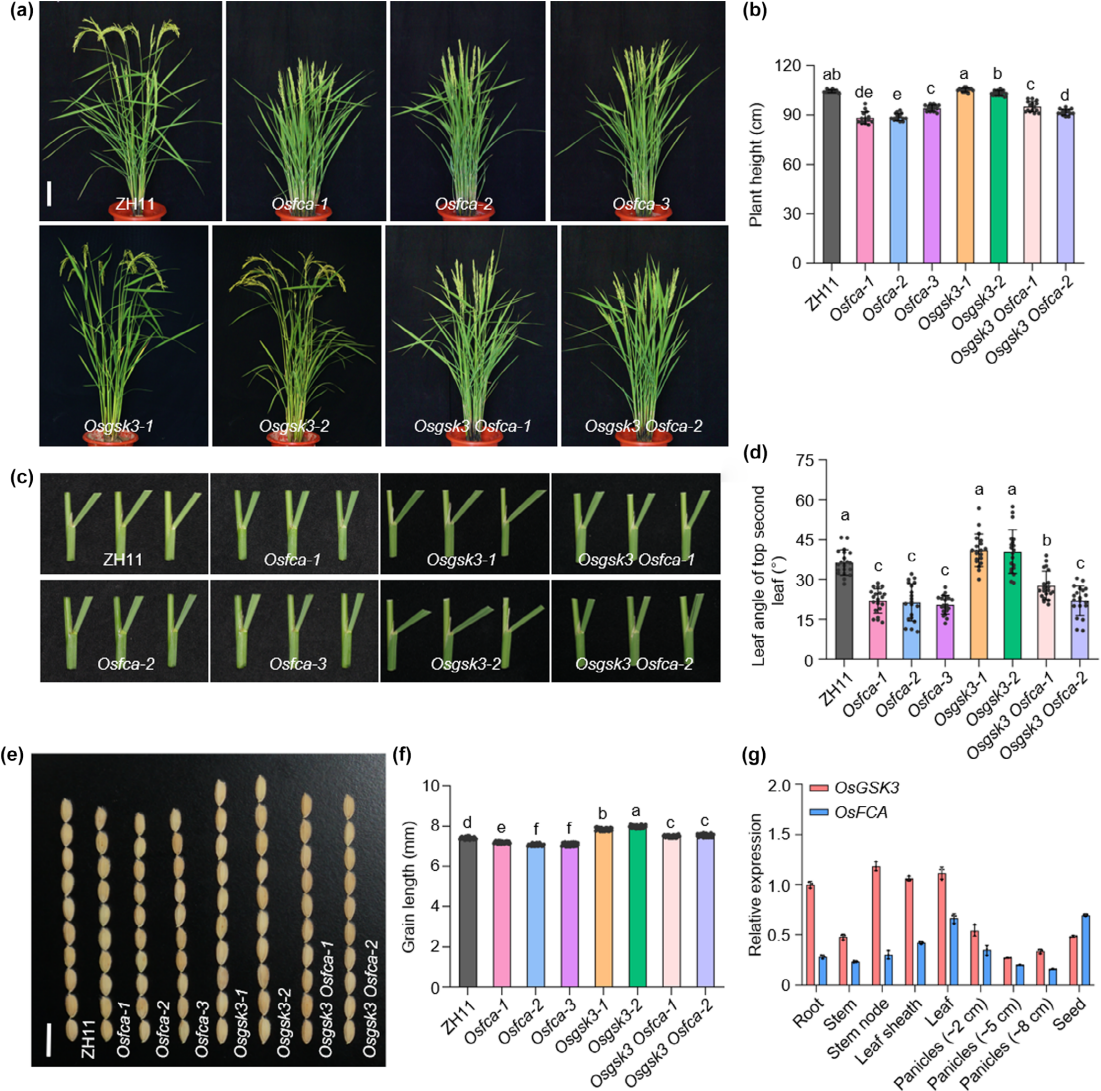
FLOWERING CONTROL LOCUS A (OsFCA) is involved in regulating rice grain shape and plant architecture development. (a) Representative profile photographs of Zhonghua11 (ZH11), *Osfca*, *Osgsk3* and *Osgsk3 Osfca* plants at the mature stage. Bar, 10 cm. (b) Statistical data on plant height of ZH11, *Osfca*, *Osgsk3* and *Osgsk3 Osfca* plants. Data are means ± SD (*n* = 15). (c) Representative photograph showing the angle of the second top leaf at the grain‐filling stage. (d) Statistical data on angle of the top second leaf at the grain‐filling stage. Data are mean ± SD (*n* = 20). (e) Grain size of ZH11, *Osfca*, *Osgsk3*, and *Osgsk3 Osfca* plants. Bar, 1 cm. (f) Statistical data on grain length of ZH11, *Osfca*, *Osgsk3*, and *Osgsk3 Osfca* plants. Data are mean ± SD (*n* = 30). (g) Expression profiles of *OsFCA* and *OsGSK3* in various rice tissues. Data are mean ± SD (*n* = 3). In (b, d, f), different lowercase letters denote a significant difference between means, as determined by Duncan's multiple range tests (*P* < 0.05).

We measured the main agronomic traits of ZH11, *Osfca‐1*, *Osfca‐2*, and *Osfca‐3* plants when grown in the field. The loss of OsFCA function led to shorter plants by 15.5%, 14.9%, and 9.8% for the three *Osfca* lines compared with the WT, indicating that OsFCA positively regulates plant height (Fig. 4a,b). Further observations of leaf angle showed that all *Osfca* alleles had narrower angles by *c*. 14.4°, 15.0°, and 15.9° compared with ZH11 (Fig. 4c,d). These results show that OsFCA positively regulates the angle of rice leaves on the main stem. We also analyzed the effect of OsFCA function on grain length: The length of grains for *Osfca‐1*, *Osfca‐2*, and *Osfca‐3* plants was shorter by 2.9%, 4.3%, and 3.9%, respectively, compared with that of ZH11 (Fig. 4e,f). These results indicate that OsFCA positively regulates grain length in rice.

An investigation of the main agronomic traits in these plants showed that the double mutants *Osgsk3 Osfca‐1* and *Osgsk3 Osfca‐2* were significantly shorter and tighter than the WT and the two single mutants *Osgsk3‐1* and *Osgsk3‐2* (Fig. 4a–d). Likewise, the seed length of *Osgsk3 Osfca‐1* and *Osgsk3 Osfca‐2* was significantly shorter than that of *Osgsk3‐1* or *Osgsk3*‐*2* and significantly longer than that of ZH11 and the *Osfca‐1*, *Osfca‐2*, and *Osfca‐3* mutants (Fig. 4e,f). These results indicate that OsFCA and OsGSK3 function in the same regulatory pathway affecting grain length, and the regulation of grain length by OsGSK3 is additive with OsFCA. To analyze the spatiotemporal expression patterns of *OsGSK3* and *OsFCA* in various tissues of rice and to further explore their genetic relationship, we analyzed the expression of both genes in roots, stems, leaves, stem nodes, young panicles, and mature seeds of the *japonica* rice cultivar NJ9108. Using their expression levels in roots as a reference, *OsGSK3* expression was downregulated in *c*. 2 cm, *c*. 5 cm, and *c*. 8 cm young panicles. By contrast, *OsFCA* expression was upregulated in *c*. 2‐cm young panicles but downregulated in *c*. 5 cm and *c*. 8 cm panicles (Fig. 4g). These findings suggest that *OsGSK3* and *OsFCA* genetically interact during the initial phase of young panicle differentiation.

### Mutation of OsFCA leads to reduced BR signaling

**Fig. 5:**
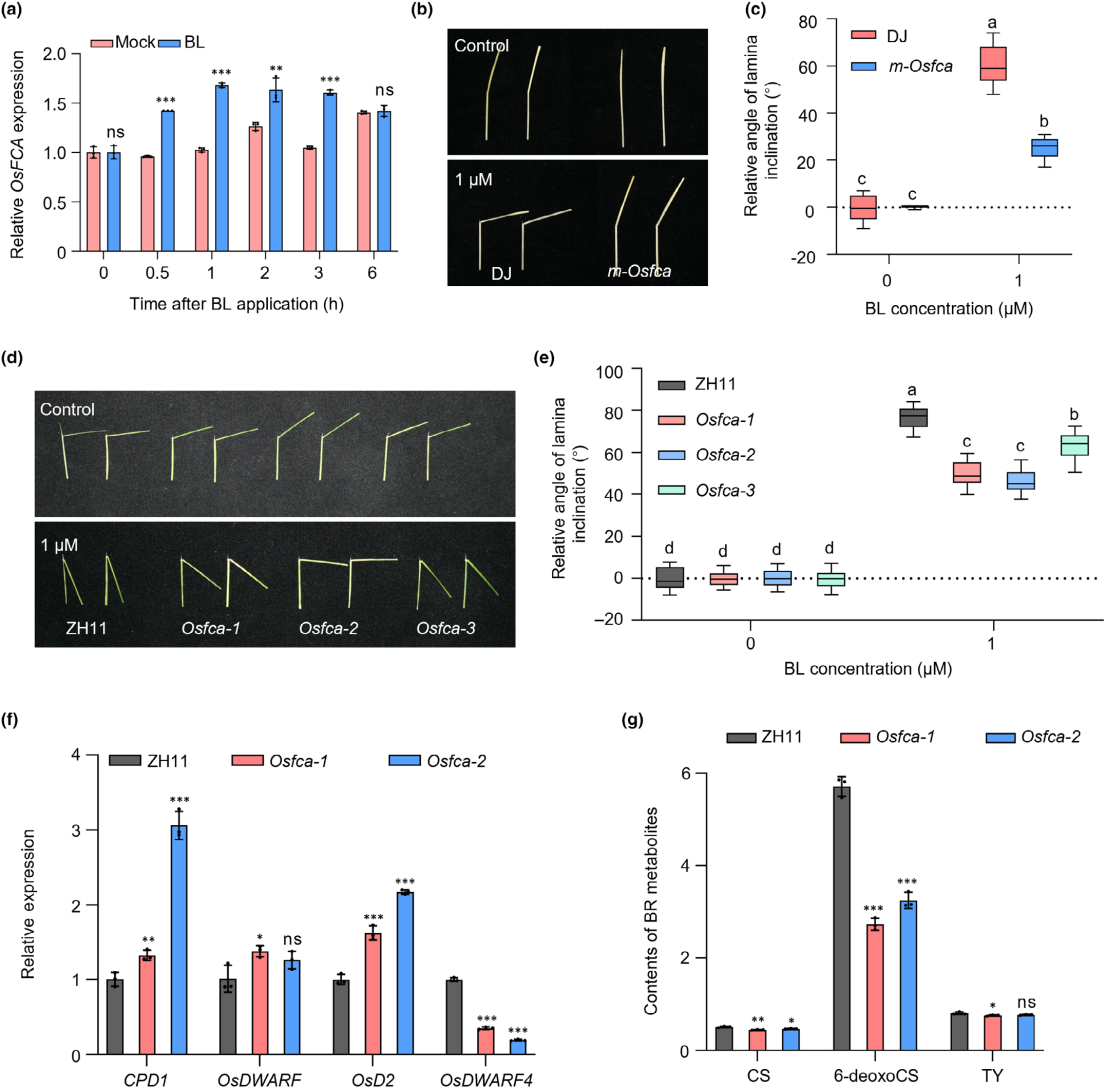
FLOWERING CONTROL LOCUS A (OsFCA) contributes to the regulation of brassinosteroid (BR) signaling. (a) Real‐time quantitative polymerase chain reaction (RT‐qPCR) analysis of *OsFCA* expression levels upon treatment with 1 μM 24‐epibrassinolide (24‐eBL). Data are mean ± SD (*n* = 3). (b) Lamina inclination assay of Dongjin (DJ) and the *m*‐*Osfca* mutant in response to treatment with 1 μM 24‐eBL. (c) Quantification of lamina inclination in DJ and the *m*‐*Osfca* mutant in response to treatment with 1 μM 24‐eBL. Data are mean ± SD (*n* = 30). (d) Lamina inclination assay of Zhonghua11 (ZH11) and the *m*‐*Osfca* mutants in response to treatment with 1 μM 24‐eBL. (e) Quantification of lamina inclination in ZH11 and the *Osfca* mutants in response to treatment with 1 μM 24‐eBL. Data are means ± SD (*n* = 30). (f) RT‐qPCR analysis of the expression levels of genes related to BR biosynthesis in ZH11 and *Osfca* mutants. Data are mean ± SD (*n* = 3). (g) Measurement of endogenous BR content in ZH11 and *Osfca* mutants. In (a, f, g), asterisks indicate significant differences compared with the mock (a) or wild‐type (f, g), as determined by a Student's *t*‐test; *, *P* < 0. 05; **, *P* < 0.01; ***, *P* < 0.001; ns, not significant. In (c, e), the horizontal line within each box indicates the median, the box bounds the interquartile range, the whiskers extend to the minimum and maximum values, and different lowercase letters denote a significant difference between mean, as determined Duncan's multiple range tests (*P* < 0.05).

We asked whether OsFCA regulates BR signaling by measuring *OsFCA* transcript levels in response to 24‐eBL treatment: Indeed, *OsFCA* transcript levels were induced by 24‐eBL treatment (Fig. 5a). When we tested the sensitivity of *m*‐*Osfca* mutants to BL treatment, we discovered that the leaf angle of DJ plants increases by *c*. 60.5°, compared with 24.9° in the *m*‐*Osfca* mutant in the DJ background, after 24‐eBL treatment, indicating that the sensitivity to 24‐eBL treatment is significantly lower in the *m*‐*Osfca* mutant than in the WT DJ (Fig. 5b,c). We obtained similar results in the ZH11 background, with the leaf angle of ZH11 increased by *c.* 76.5° after treatment with 24‐eBL, while the leaf angle of the *Osfca‐1*, *Osfca‐2*, and *Osfca‐3* mutants only increased by *c.* 49.8°, 46.3°, and 63.5°, respectively, under the same conditions, indicating that the *Osfca* mutants are significantly less sensitive to 24‐eBL treatment than ZH11 (Fig. 5d,e). These results suggest that OsFCA participates in the regulation of BR signaling in rice. RT‐qPCR analysis detected higher transcript levels for the BR biosynthesis genes *CPD1*, *OsDWARF*, and *OsD2* in *Osfca* plants relative to those in ZH11; the transcript levels of *OsDWARF4* were much lower in the mutants compared with those in the WT (Fig. 5f). These results indicate that the loss of OsFCA function affects the expression of BR biosynthesis genes. To this end, we quantified endogenous BR levels and found that both castasterone (CS) and 6‐deoxocastasterone (6‐deoxoCS) were reduced in the *Osfca* mutants compared to ZH11 (Fig. 5g). These data support the conclusion that loss of *OsFCA* impairs both BR signaling and BR biosynthesis.

When we tested the sensitivity of leaf angle to BL treatment in the *Osfca* and *Osgsk3* single and double mutants, the leaf angle of treated *Osfca‐1* plants was only *c*. 49.8° larger, and that of *Osgsk3 Osfca‐2* plants was only *c*. 58.7° larger after 24‐eBL treatment. The leaf angle of *Osgsk3‐2* plants reached *c*. 86.0° following treatment with 24‐eBL, a value significantly higher than that seen in ZH11 (Fig. S6a,b). Thus, the sensitivity of leaf angle to BL treatment was significantly higher in *Osgsk3‐2* and significantly lower in *Osfca‐1* and *Osgsk3 Osfca‐2* than in the WT.

### Transcriptome analysis identifies genome‐wide expression changes regulated by OsFCA in young rice panicles

**Fig. 6:**
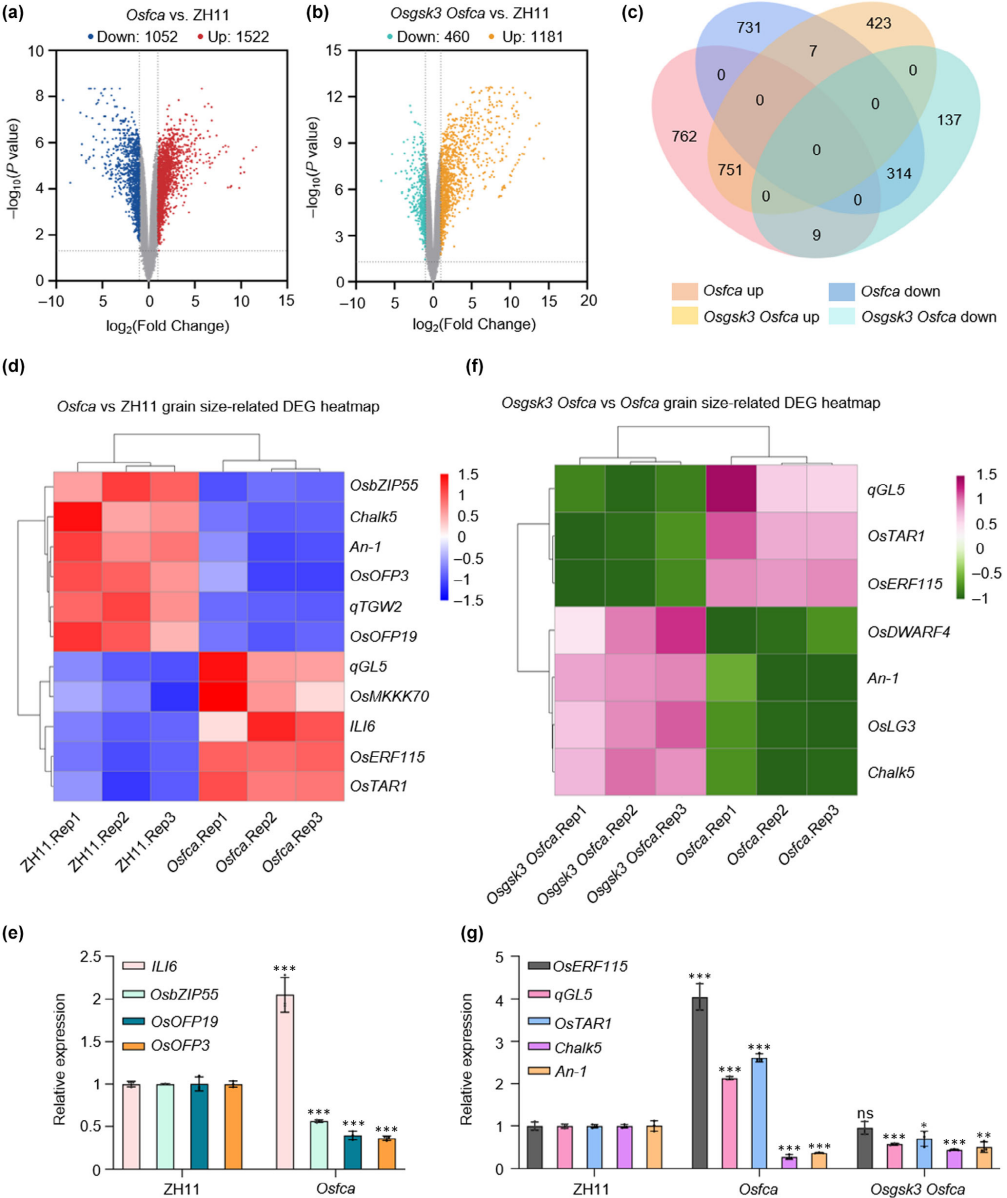
Analysis of the differentially expressed genes (DEGs) in *Osfca* and *Osgsk3 Osfca* mutants compared to Zhonghua11 (ZH11). (a) Volcano plots of total gene expression profiles in *Osfca* compared with ZH11. DEGs were defined by absolute log_2_(Fold Change) > 1 and *P* value < 0.05. (b) Volcano plots of total gene expression profiles in *Osgsk3 Osfca* compared with ZH11. DEGs were defined by absolute log_2_(Fold Change) > 1 and *P* value < 0.05. (c) Venn diagram showing the overlap between the DEGs in *Osfca* and *Osgsk3 Osfca* compared to ZH11. (d) Cluster heatmap of the grain size‐related regulatory genes screened from DEGs in young panicles of *Osfca* and ZH11 with three biological replicates per genotype. Scale bar shows fold changes, values are normalized by z‐score scheme, red and blue colors indicate up‐ and downregulated, respectively. (e) Relative transcript levels of four grain size‐related regulatory genes in ZH11 and *Osfca* by reverse transcription quantitative polymerase chain reaction (RT‐qPCR). Data are mean ± *SD* (*n* = 3). (f) Cluster heatmap of the grain size‐related regulatory genes screened from DEGs in young panicles of *Osgsk3 Osfca* and *Osfca* with three biological replicates per genotype. Scale bar shows fold changes, values are normalized by z‐score scheme, rose red and green colors indicate up‐ and downregulated, respectively. (g) Relative transcript levels of five grain size‐related regulatory genes in ZH11, *Osfca* and *Osgsk3 Osfca* by RT‐qPCR. Data are mean ± *SD* (*n* = 3). In (e, g), asterisks indicate significant differences compared with the wild‐type, as determined by a Student's *t*‐test; *, *P* < 0. 05; **, *P* < 0.01; ***, *P* < 0.001; ns, not significant.

To elucidate the molecular mechanism underlying OsFCA‐mediated regulation of grain development in rice, we performed RNA sequencing (RNA‐seq) analysis using young panicles to characterize the regulatory pattern of OsFCA during rice grain development. A total of 2574 genes were differentially expressed between ZH11 and *Osfca* (Fold change >2, *P* value <0.05), comprising 1522 upregulated and 1052 downregulated genes (Figs 6a, S7a). Compared with ZH11, the *Osgsk3 Osfca* double mutant showed a total of 1641 DEGs, including 1181 upregulated and 460 downregulated ones (Figs 6b, S7b). Venn analysis revealed 1081 commonly regulated genes, while 1493 and 560 genes were uniquely regulated in the *Osfca* and *Osgsk3 Osfca* genotypes, respectively (Fig. 6c), thus supporting the coexistence of shared and genotype‐specific transcriptional regulatory modules.

Among the DEGs, grain size‐related regulatory genes were screened and validated by RT‐qPCR. The results showed that *ILI6* displayed elevated expression in *Osfca*, whereas transcript levels of *OsbZIP55*, *OsOFP19*, and *OsOFP3* were significantly reduced in this mutant (Fig. 6d,e). In addition, compared with *Osfca*, seven grain size‐related genes showed differential expression in *Osgsk3 Osfca* (Fig. 6f). RT‐qPCR validation revealed that *qGL5* and *OsTAR1* were upregulated in *Osfca* but downregulated in *Osgsk3 Osfca*, whereas *Chalk5* and *An‐1* were downregulated in both *Osfca* and *Osgsk3 Osfca* (Fig. 6g). These results imply that *Chalk5* and *An‐1* may function as downstream targets of the OsGSK3‐OsFCA module in regulating rice grain size development.

### OsFCA regulates rice heading date

**Fig. 7:**
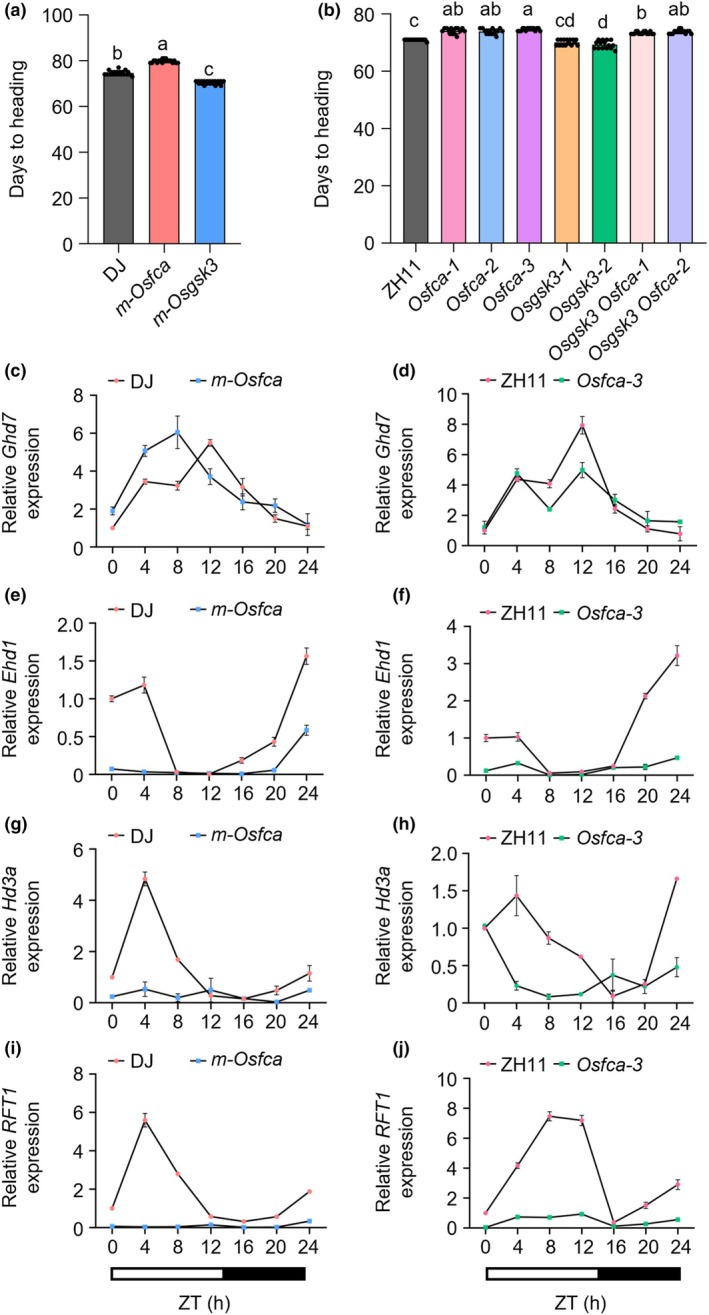
FLOWERING CONTROL LOCUS A (OsFCA) regulates heading date in rice. (a) Heading date of Dongjin (DJ) and the T‐DNA insertion mutants *m*‐*Osfca* and *m*‐*Osgsk3*. Data are mean ± SD (*n* = 20). (b) Heading date of Zhonghua11 (ZH11), *Osfca*, *Osgsk3* and *Osgsk3 Osfca* plants. Data are mean ± SD (*n* = 15). (c, e, g, i) Real‐time quantitative polymerase chain reaction (RT‐qPCR) analysis of *Grain number, plant height, and heading date 7* (*Ghd7*) (c), *Early heading date 1* (*Ehd1*) (e), *Heading date 3a* (*Hd3a*) (g), and *RICE FLOWERING LOCUS T 1* (*RFT1*) (i) expression levels over one diurnal cycle in DJ and *m‐Osfca* plants. Data are mean ± SD (*n* = 3). ZT, Zeitgeber time. (d, f, h, j) RT‐qPCR analysis of *Ghd7* (d), *Ehd1* (f), *Hd3a* (h), and *RFT1* (j) expression levels over one diurnal cycle in ZH11 and *Osfca‐3* plants. Data are mean ± SD (*n* = 3). ZT, Zeitgeber time. In (a, b), different lowercase letters denote a significant difference between means, as determined by Duncan's multiple range tests (*P* < 0.05).

Phenotypic observations of field‐grown plants at Nanjing Baima Teaching and Research Base revealed the delayed heading of *m*‐*Osfca* and *Osfca* mutants relative to their respective WTs. Based on field data collected in 2022, the heading date of the *m*‐*Osfca* mutant was 5.2 d later than that of DJ (Fig. 7a). We obtained congruent results in the ZH11 background, with the heading dates of the *Osfca‐1*, *Osfca‐2* and *Osfca‐3* mutants being 3.1 d, 2.9 d, and 3.7 d later than that of ZH11 (Fig. 7b), respectively, indicating that OsFCA regulates the heading stage in rice. To explore the regulatory relationship between OsGSK3 and heading in rice in more detail, we measured the heading date of *m‐Osgsk3*. The *m‐Osgsk3* mutant flowered earlier than DJ, with the heading date of the *m‐Osgsk3* mutant being 4.2 d earlier than that of DJ, and the heading date of *Osgsk3‐1* and *Osgsk3‐2* was 0.9 and 1.7 d earlier than that of ZH11 (Fig. 7a,b), suggesting that OsGSK3 contributes to the regulation of rice heading date through phosphorylation of OsFCA. At the same time, the heading date of *Osgsk3 Osfca‐1* and *Osgsk3 Osfca‐2* was 2.4 and 2.9 d later than that of ZH11 (Fig. 7b). These results indicate that OsFCA acts downstream of OsGSK3 in the regulation of heading stage. We assessed the diurnal expression patterns of heading stage‐related genes by RT‐qPCR in DJ and *m*‐*Osfca*, and separately in ZH11 and *Osfca‐3*, when entrained to long‐day conditions (14 h : 10 h, light : dark). Initially, we observed a circadian oscillation in *OsFCA* transcription, with transcript levels gradually increasing during light exposure, peaking at ZT12, and subsequently declining upon the onset of darkness (Fig. S8). Loss of OsFCA function in the DJ background led to an earlier peak in *Grain number*, *plant height*, *and heading date 7* (*Ghd7*) transcript levels from ZT12 to ZT8 (Fig. 7c,d). The transcript levels of *Early heading date 1* (*Ehd1*), *Heading date 3a* (*Hd3a*), and *RICE FLOWERING LOCUS T 1* (*RFT1*) were much lower in the mutants relative to their respective WTs (Fig. 7e–j). These data indicate that OsFCA promotes heading under long‐day conditions by repressing *Ghd7* expression and activating *Ehd1*, *Hd3a*, and *RFT1*.

### OsGSK3 phosphorylates and inhibits phase separation of OsFCA at Ser‐43 and Ser‐45

**Fig. 8:**
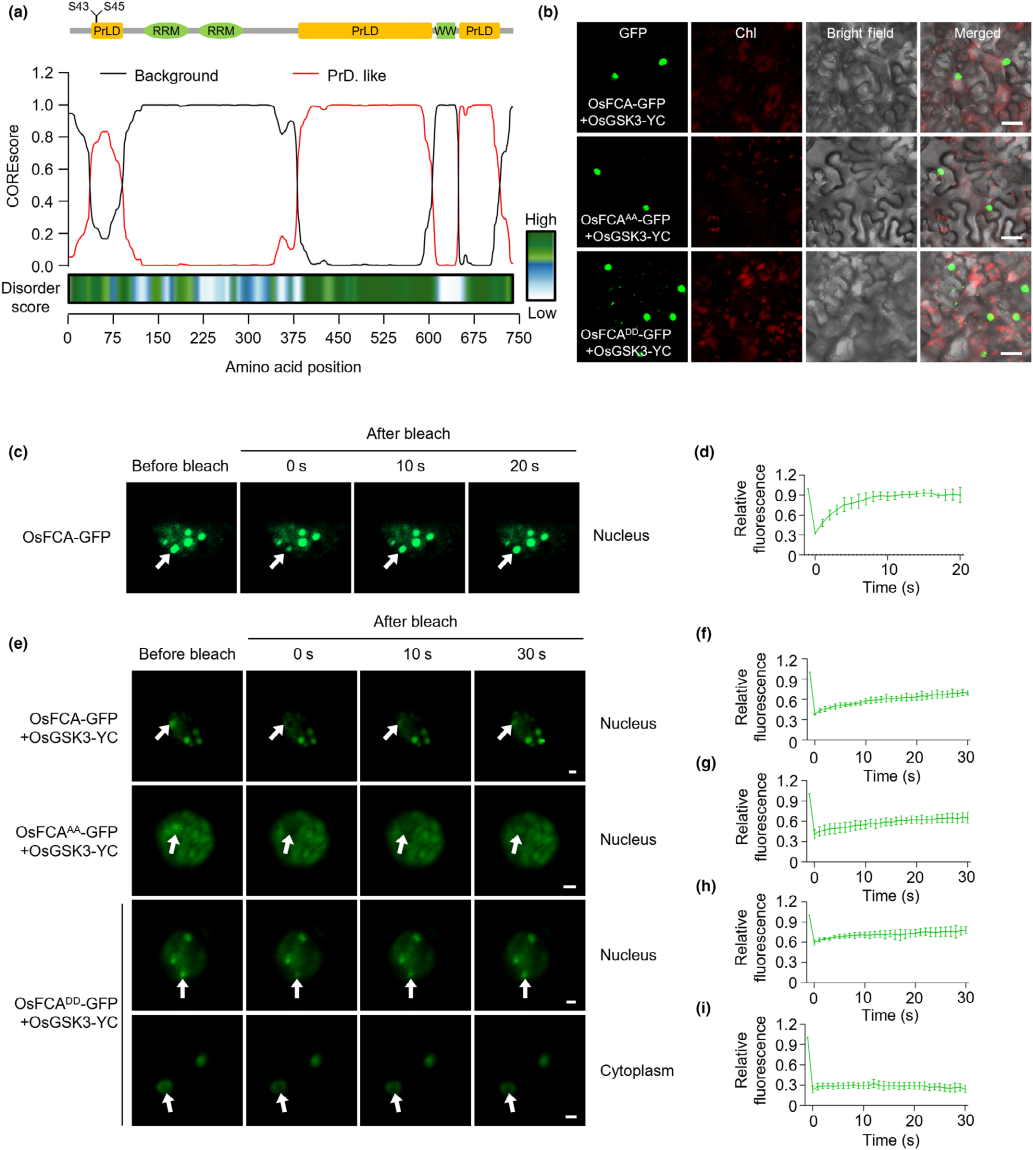
FLOWERING CONTROL LOCUS A (OsFCA) protein undergoes phase separation *in vivo*. (a) Upper panel, schematic diagram of the OsFCA protein. PrLD, prion‐like domain; RRM, RNA recognition motif; WW, domain with two conserved Trp (W) residues. Middle, prediction of PrLDs via ‘Prion‐like Amino Acid Composition’ (PLAAC, http://plaac.wi.mit.edu/). Lower panel, prediction of disordered regions via D^2^P^2^ (http://d2p2.pro/). (b) Subcellular localization of OsFCA‐GFP, OsFCA^AA^‐GFP, and OsFCA^DD^‐GFP following cotransfection with OsGSK3‐YC. GFP, green fluorescent protein; Chlorophyll, Chl autofluorescence; Merged, merged fields. Bars, 30 μm. (c) Fluorescence recovery after photobleaching (FRAP) of OsFCA‐GFP droplets in the nucleus of tobacco cells. Time 0 denotes the moment of the photobleaching pulse. The white arrow indicates the photobleached regions for recovery analysis. Bar, 2 μm. (d) Time course of relative fluorescence intensity recovery of OsFCA droplets after photobleaching. (e) FRAP of OsFCA‐GFP, OsFCA^AA^‐GFP, and OsFCA^DD^‐GFP droplets in the nucleus or cytoplasm of tobacco cells when co‐expression with OsGSK3‐YC. Time 0 denotes the moment of the photobleaching pulse. The white arrow indicates the photobleached regions for recovery analysis. Bars, 2 μm. (f–i) Time course of relative fluorescence intensity recovery of OsFCA droplets after photobleaching, in tobacco cell nucleus cotransformed with OsFCA‐GFP and OsGSK3‐YC (f), in tobacco cell nucleus cotransformed with OsFCA^AA^‐GFP and OsGSK3‐YC (g), in tobacco cell nucleus cotransformed with OsFCA^DD^‐GFP and OsGSK3‐YC (h), and in tobacco cell cytoplasm cotransformed with OsFCA^DD^‐GFP and OsGSK3‐YC (i).

OsFCA encodes a putative RBP harboring two RNA‐binding motifs and one WW domain (Fig. 8a). The Prion‐like Amino Acid Composition (PLAAC) prediction tool identified three candidate prion‐like domains (PrLDs), while the D^2^P^2^ database revealed four intrinsically disordered regions, three of which overlap substantially with the predicted PrLDs, indicating a modular architecture conducive to phase separation (Fig. 8a). To investigate how OsGSK3‐mediated phosphorylation of OsFCA affects its function, we first examined whether OsGSK3 influences the phase separation of OsFCA. We investigated the functional consequences of OsFCA phosphorylation by mutating the two phosphorylation sites detected in the phosphoproteomics and *in vitro* phosphorylation analysis (Fig. S9). Accordingly, we changed the residues S43 and S45 to alanine (Ala, A) to mimic a nonphosphorylatable protein (OsFCA^AA^) or to aspartic acid (Asp, D) to mimic the phosphorylated state (OsFCA^DD^). Co‐expression of OsGSK3 with OsFCA, OsFCA^AA^, and OsFCA^DD^ in tobacco epidermal cells revealed that OsGSK3 co‐expression with OsFCA^DD^ led to the appearance of liquid droplet‐like condensates in the cytoplasm (Fig. 8b). These data prompted us to perform fluorescence recovery after photobleaching (FRAP) experiments to investigate whether OsFCA undergoes phase separation. The results showed that OsFCA condenses into droplets in the nucleus and gradually recovers fluorescence after photobleaching (Fig. 8c,d), indicating that phase separation can occur in the nucleus.

FRAP analysis further revealed that when co‐expressed with OsGSK3‐YC, the nucleus‐localized OsFCA‐GFP, OsFCA^AA^‐GFP, and OsFCA^DD^‐GFP were capable of gradual fluorescence recovery after photobleaching (Fig. 8e–h). Meanwhile, the phosphorylated form OsFCA^DD^‐GFP exhibited a shift in localization to the cytoplasm, and its fluorescence failed to recover after photobleaching (Fig. 8e,i), suggesting that phosphorylated OsFCA^DD^ loses its ability to perform regulatory functions via phase separation upon nuclear export.

**Fig. 9:**
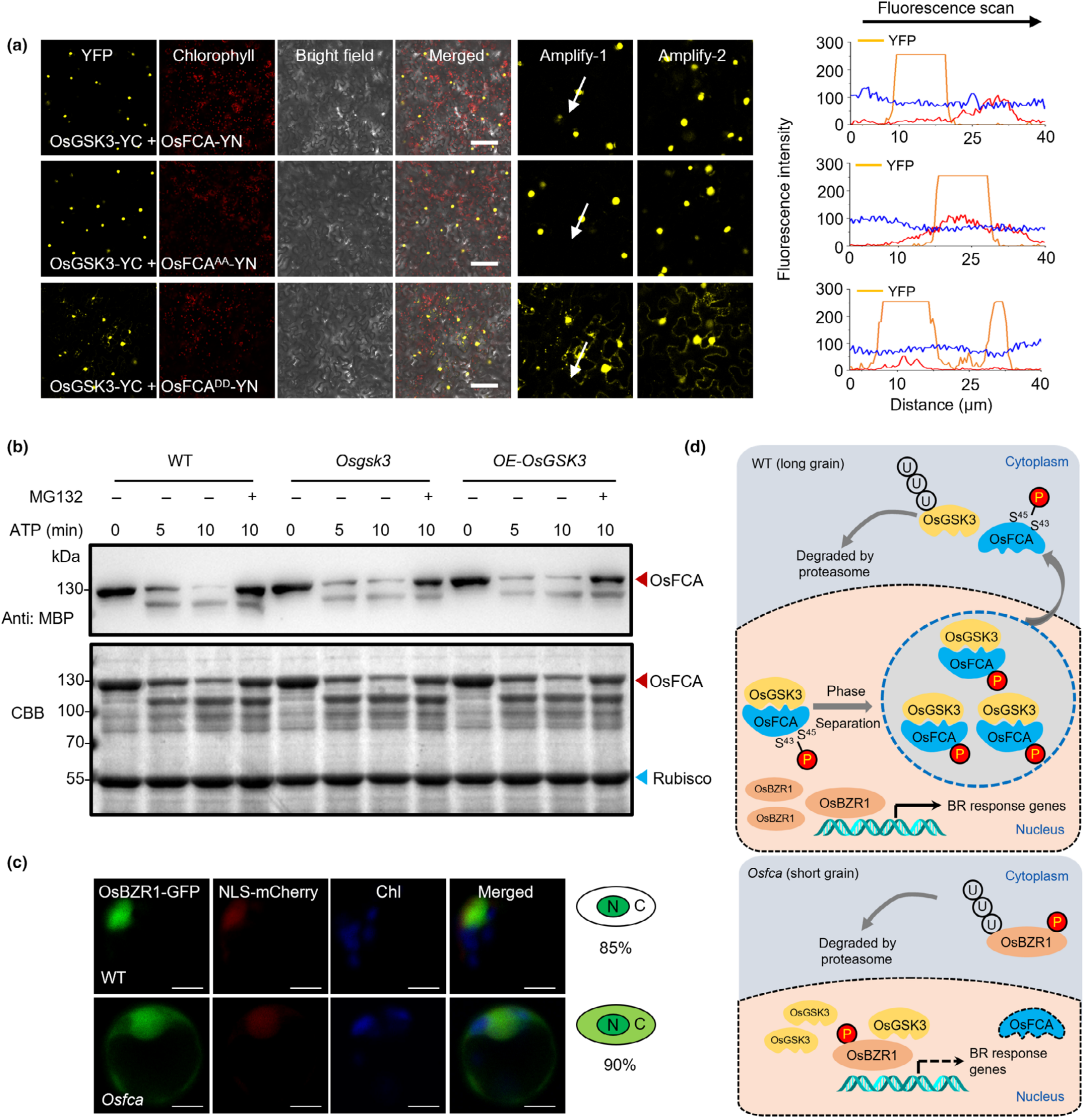
Phosphorylation promotes the translocation of OsGSK3‐OsFCA from the nucleus to the cytoplasm. (a) Bimolecular fluorescence complementation assay of the interaction between OsFCA^AA^ or OsFCA^DD^ and OsGSK3 in the epidermal cells of *Nicotiana benthamiana* leaves. YFP, yellow fluorescent protein; Chlorophyll, Chl autofluorescence; Merged, merged fields. Bars, 100 μm. The white arrows indicate the fluorescence scanning direction. (b) Cell‐free protein degradation assays were conducted to analyze the stability of OsFCA in total protein extract from wild‐type (WT), *Osgsk3*, and *OE*‐*OsGSK3* plants. Anti‐MBP antibody was used to detect changes in OsFCA protein levels. Coomassie brilliant blue staining served as the loading control. The position of the MBP‐OsFCA bands is indicated by a red arrow, and the position of the Rubisco band is marked with a blue arrow. (c) Subcellular localization of OsBZR1 in the rice protoplasts. The GFP fluorescence signals, mCherry fluorescence signals, and autofluorescence signals from chloroplasts were pseudo‐colored as green, red, and blue, respectively. Bars, 3 μm. (d) A working model for OsGSK3‐mediated phosphorylation of OsFCA in the regulation of brassinosteroid (BR) signaling and rice growth and development. In the nucleus, OsFCA forms phase‐separated condensates that sequester OsGSK3, shielding OsBZR1 from phosphorylation; once phosphorylated, the complex is exported to the cytoplasm where OsGSK3 is degraded. They mutually influence each other and are involved in regulating rice grain size formation. Solid black arrows indicate normal expression of BR‐responsive genes, while dashed black arrows indicate reduced expression of BR‐responsive genes.

It is known that co‐expression of OsGSK3 and OsFCA affects the subcellular localization of OsFCA via phase separation; whether the localization of their PPI also changes remains unclear. To address this, we performed a BiFC assay to test whether the above phosphorylation site mutations affect the interaction between OsFCA and OsGSK3. We determined that OsFCA^AA^ interacts with OsGSK3 in *N. benthamiana* leaf epidermal cells, with fluorescence signals detected in the nucleus. By contrast, OsFCA^DD^ and OsGSK3 interacted in the nucleus and cytoplasm (Fig. 9a). We therefore hypothesize that phosphorylation of OsFCA promotes its translocation from the nucleus to the cytoplasm, a change that requires its phosphorylation by OsGSK3. To investigate whether the phosphorylation of OsFCA by OsGSK3 affects the degradation of OsFCA, we conducted a cell‐free degradation assay. The results demonstrated that OsFCA undergoes degradation via the ubiquitin‐proteasome pathway (Fig. 9b). However, compared with the WT, the degradation rates of OsFCA showed no significant changes in the *Osgsk3* and *OE‐OsGSK3* materials (Fig. 9b). These findings indicate that phosphorylation of OsFCA by OsGSK3 does not affect its degradation.

Previous studies have shown that OsGSK3 phosphorylates OsBZR1 in the nucleus, thereby regulating the nucleocytoplasmic distribution of OsBZR1 (Gao *et al*., 2019). Therefore, we further investigated whether the interaction between OsFCA and OsGSK3 affects the function of OsBZR1. Analysis of OsBZR1 subcellular localization in WT ZH11 and *Osfca* mutants revealed that in the absence of OsFCA, OsBZR1 exhibits increased translocation from the nucleus to the cytoplasm (Fig. 9c). This indicates that OsFCA regulates OsGSK3 distribution through phase separation, thereby mediating OsGSK3‐dependent nucleocytoplasmic distribution of OsBZR1, which ultimately modulates BR signaling.

## Discussion

In this study, we performed phosphoproteomics to identify OsFCA controlling flowering time as a phosphorylation substrate of OsGSK3, a negative regulator of grain length. OsFCA dysfunction resulted in a series of BR‐deficient phenotypes, including shorter plant height, narrower leaf angles, and shorter grains. At the protein level, BR signaling inhibits the phosphorylation of DLT by OsGSK2, allowing the accumulation of active DLT in its dephosphorylated form; at the transcriptional level, BR imposes OsBZR1‐mediated transcriptional inhibition (Tong *et al*., 2009, 2012). In contrast to the inhibitory effect on *DLT* transcription by OsBZR1, DLT promotes the transcription of *OsBZR1*, creating a negative feedback loop that regulates the expression of BR biosynthesis genes. DLT can also interact with OSH15 to promote the transcriptional activation of the BR signaling receptor gene *OsBRI1* (Tong *et al*., 2009; Niu *et al*., 2022). The *m*‐*Osfca* loss‐of‐function mutants showed a less sensitive phenotype upon BL treatment. The transcript levels of BR biosynthesis genes *CPD1*, *OsDWARF*, and *OsD2* were higher, while those of *OsDWARF4* were lower.

We showed here that OsFCA interacts with and is phosphorylated by OsGSK3, a negative regulator of BR signaling. We turned to CRISPR/Cas9‐mediated gene editing to generate *Osgsk3 Osfca* double mutants. An investigation of major agronomic traits showed that the *Osgsk3 Osfca* plants were significantly shorter than the WT and *Osgsk3* plants, while the *Osgsk3 Osfca* grains were significantly shorter than those of *Osgsk3* and significantly longer than those of ZH11 and *Osfca*. The sensitivity of *Osgsk3 Osfca‐2* leaf angles to BL treatment was significantly greater than that of *Osfca‐1* and significantly weaker than that of *Osgsk3‐2*. These results indicate that OsFCA and OsGSK3 jointly mediate BR signaling to regulate grain length in rice, and the regulatory effects of OsGSK3 on the BR signal transduction pathway and grain length are partially dependent on OsFCA.

Heading date is a key agronomic trait that determines the rice planting season and its regional adaptability, as well as yield. Early maturation or late heading can lead to lower yields due to an insufficient growth period in the vegetative stage or insufficient grain‐filling and maturity time (Song *et al*., 2015). OsFCA is the rice ortholog of Arabidopsis FCA, a component of the autonomous flowering time pathway; the phosphorylation levels of OsFCA in *m‐Osgsk3* mutant or in BL‐treated DJ samples were significantly lower than those in DJ, indicating that impaired OsGSK3 function resulted in lower phosphorylation levels of OsFCA. When we simultaneously mutated the two phosphorylation sites S43 and S45 of OsFCA detected in the phosphoproteomic analysis to a nonphosphorylatable state (OsFCA^AA^) or a constitutively phosphorylated state (OsFCA^DD^), we observed the phosphorylation status of OsFCA^DD^ did facilitate the transfer of its interaction with OsGSK3 from the nucleus to the cytoplasm, and we speculate that OsFCA can become cytoplasm‐localized after phosphorylation, and this change in localization requires OsGSK3 and unknown protein kinases.

In this study, we showed that the loss of OsFCA function severely disrupts the normal diurnal expression pattern of *Ehd1* under long‐day conditions, and strongly represses the transcript accumulation of the florigens *Hd3a* and *RFT1*, resulting in an earlier peak in the transcript levels of the heading repressor gene *Ghd7* upstream of *Ehd1. The* results of this study indicate that OsFCA regulates heading date in rice grown under long‐day conditions by inhibiting *Ghd7* expression and activating the expression of *Ehd1*, *Hd3a*, and *RFT1*. OsBZR1 binds to the *Ghd7* promoter and inhibits its transcription in rice, activating the downstream heading regulatory pathway by recruiting the histone deacetylase HDA703 to synergistically mediate the transcriptional repression of *Ghd7* with OsBZR1 (Wang *et al*., 2020). Mutations in the cytochrome P450 gene *OsCYP51H3*, encoding a key enzyme in BR biosynthesis, result in a late‐flowering phenotype (Jiao *et al*., 2022). The T‐DNA insertion mutant *m‐Osgsk3* flowered earlier than its WT. We speculate that the direct regulation of OsFCA by OsGSK3 affects OsFCA‐mediated heading time.

Based on these findings, we propose a hypothesis: Phosphorylation of OsFCA by OsGSK3 can modulate the regulatory function of OsFCA by influencing its subcellular localization. Additionally, the condensates formed by phosphorylated OsFCA facilitate the translocation of OsGSK3 from the nucleus to the cytoplasm, thereby promoting its degradation in the cytoplasm. Here, we propose a model: qGL3 dephosphorylates and stabilizes OsGSK3, a key negative regulator of BR signaling. OsGSK3 is capable of autophosphorylation. In the *Osfca* mutant, the phosphorylated form of OsFCA fails to bring OsGSK3 into the cytoplasm, leading to the accumulation of OsGSK3 in the nucleus. In the nucleus, OsGSK3 phosphorylates OsBZR1 and promotes its cytoplasmic localization, thereby reducing grain length. In wild‐type plants, OsGSK3 interacts with OsFCA in the nucleus and forms biomolecular condensates through LLPS. OsGSK3 phosphorylates OsFCA, and the phosphorylated OsFCA facilitates the co‐translocation of OsGSK3 to the cytoplasm. After entering the cytoplasm, OsGSK3 and OsFCA no longer maintain the condensate state. Previous studies have shown that cytoplasmic OsGSK3 can be degraded via the ubiquitin‐proteasome pathway. Although OsGSK3 affects the phosphorylation status of OsFCA, it does not alter its degradation rate, indicating that OsGSK3‐mediated phosphorylation primarily regulates the nucleocytoplasmic distribution of OsFCA. The condensates formed by OsFCA and OsGSK3 in the nucleus attenuate the function of OsBZR1 by OsGSK3. In addition, OsBZR1 modulates the expression of BR‐responsive genes; consequently, compared with the *Osfca* mutant, wild‐type plants exhibit longer grains. Overall, OsFCA interacts with OsGSK3 in the nucleus and forms condensates via LLPS. Then, the phosphorylated OsFCA promotes the translocation of the OsGSK3‐OsFCA complex into the cytoplasm, and OsGSK3 and OsFCA no longer exist in a condensate state. Therefore, the condensation of OsFCA and OsGSK3 in the nucleus protects OsBZR1. In the cytoplasm, the dissolution of the condensate between OsGSK3 and OsFCA facilitates the degradation of OsGSK3 without affecting the degradation of OsFCA (Fig. 9d). This mechanism provides precise regulation of grain length in rice.

Collectively, our study argues that the OsGSK3‐OsFCA module is a key regulator in rice BR signal transduction, grain length control, and flowering time modulation. This finding enriches the rice BR signaling network and provides a theoretical foundation for utilizing OsGSK3 and OsFCA in molecular breeding of rice.

## Competing interests

None declared.

## Author contributions

XG, JZ and JH conceived the study, designed the experiments and analyzed the data. JZ, SZ, QY, QD, JL, XW, HD, YS, RM, YJ and JC performed the experiments. FW analyzed the omic data. JZ and XG wrote the manuscript. XG, JH and HZ supervised the project.

## Disclaimer

The New Phytologist Foundation remains neutral with regard to jurisdictional claims in maps and in any institutional affiliations.

## Supporting information


**Fig. S1** Cluster heatmap of proteins showing consistent upregulation or downregulation trends across treatments.
**Fig. S2** Y2H analysis of interactions between OsGSK3 and candidates from the phosphoproteome.
**Fig. S3** Phenotypes of the *m‐Osfca* mutant.
**Fig. S4** Identification of *Osfca* mutants.
**Fig. S5** Identification of *Osgsk3* and *Osgsk3 Osfca* mutants.
**Fig. S6** OsFCA contributes to OsGSK3‐mediated BR signal transduction.
**Fig. S7** Cluster heatmap of all DEGs in ZH11, *Osfca*, and *Osgsk3 Osfca*.
**Fig. S8** *OsFCA* transcription exhibits a circadian rhythmic.
**Fig. S9** Identification of OsFCA phosphorylation sites.
**Table S1** Primers used in this study.
**Table S2** Protein groups identified.
**Table S3** Unique peptides identified.
**Table S4** Phosphopeptides identified.
**Table S5** Phosphorylation sites identified.
**Table S6** Quantifiable protein entries.
**Table S7** Representative phosphorylation sites.
**Table S8** Proteins associated with OsGSK3 and BR signaling.
**Table S9** Proteins forming the protein–protein interaction (PPI) network with OsGSK3 as the central node.
Please note: Wiley is not responsible for the content or functionality of any Supporting Information supplied by the authors. Any queries (other than missing material) should be directed to the *New Phytologist* Central Office.

## Data Availability

RNA‐seq data of ZH11, *Osfca*, and *Osgsk3 Osfca* plants in this study are available at NCBI under the GSE332753. Phosphoproteomic data supporting this study are available in the supplementary data that accompanies this article (Figs S1–S9; Tables S1–S9). Sequence data from this article have been deposited in GenBank/EMBL under the following accession nos.: *OsFCA*, LOC_Os09g03610; *OsGSK3*, LOC_Os02g14130; *CPD1*, LOC_Os11g04710; *OsDWARF*, LOC_Os03g40540; *OsDWARF4*, LOC_Os03g12660; *OsD2*, LOC_Os01g10040; *Hd3a*, LOC_Os06g06320; *Ehd1*, LOC_Os10g32600; *Ghd7*, LOC_Os07g15770; *RFT1*, LOC_Os06g06300; *OsERF115*, LOC_Os08g41030; *qGL5*, LOC_Os05g37470; *OsTAR1*, LOC_Os05g07720; *ILI6*, LOC_Os03g07510; *Chalk5*, LOC_Os05g06480; *An‐1*, LOC_Os04g28280; *OsbZIP55*, LOC_Os06g50600; *OsOFP19*, LOC_Os05g25910; *OsOFP3*, LOC_Os01g53160; *SDG725*, LOC_Os02g34850; *OsActin*, LOC_Os03g50885.

## References

[nph71320-bib-0001] Attia K , Li KG , Wei C , He GM , Su W , Yang JS . 2005. Transformation and functional expression of the *rFCA‐RRM2* gene in rice. Journal of Integrative Plant Biology 47: 823–830.

[nph71320-bib-0002] Banani SF , Lee HO , Hyman AA , Rosen MK . 2017. Biomolecular condensates: organizers of cellular biochemistry. Nature Reviews. Molecular Cell Biology 18: 285–298. 28225081 10.1038/nrm.2017.7 PMC7434221

[nph71320-bib-0003] Blackman BK . 2017. Changing responses to changing seasons: Natural variation in the plasticity of flowering time. Plant Physiology 173: 16–26. 27872243 10.1104/pp.16.01683 PMC5210766

[nph71320-bib-0004] Brangwynne CP , Eckmann CR , Courson DS , Rybarska A , Hoege C , Gharakhani J , Jülicher F , Hyman AA . 2009. Germline P granules are liquid droplets that localize by controlled dissolution/condensation. Science 324: 1729–1732. 19460965 10.1126/science.1172046

[nph71320-bib-0005] Cui S , Song P , Wang C , Chen S , Hao B , Xu Z , Cai L , Chen X , Zhu S , Gan X *et al*. 2024. The RNA binding protein EHD6 recruits the m^6^A reader YTH07 and sequesters OsCOL4 mRNA into phase‐separated ribonucleoprotein condensates to promote rice flowering. Molecular Plant 17: 935–954. 38720462 10.1016/j.molp.2024.05.002

[nph71320-bib-0006] Fan S , Zhang Y , Zhu S , Shen L . 2024. Plant RNA‐binding proteins: Phase separation dynamics and functional mechanisms underlying plant development and stress responses. Molecular Plant 17: 531–551. 38419328 10.1016/j.molp.2024.02.016

[nph71320-bib-0007] Fang X , Wang L , Ishikawa R , Li Y , Fiedler M , Liu F , Calder G , Rowan B , Weigel D , Li P *et al*. 2019. Arabidopsis FLL2 promotes liquid‐liquid phase separation of polyadenylation complexes. Nature 569: 265–269. 31043738 10.1038/s41586-019-1165-8 PMC6625965

[nph71320-bib-0008] Gao X , Zhang JQ , Zhang X , Zhou J , Jiang Z , Huang P , Tang Z , Bao Y , Cheng J , Tang H *et al*. 2019. Rice qGL3/OsPPKL1 functions with the GSK3/SHAGGY‐like kinase OsGSK3 to modulate brassinosteroid signaling. Plant Cell 31: 1077–1093. 30923230 10.1105/tpc.18.00836 PMC6533024

[nph71320-bib-0009] Gaudinier A , Blackman BK . 2020. Evolutionary processes from the perspective of flowering time diversity. New Phytologist 225: 1883–1898. 31536639 10.1111/nph.16205

[nph71320-bib-0010] Gendron JM , Liu JS , Fan M , Bai MY , Wenkel S , Springer PS , Barton MK , Wang ZY . 2012. Brassinosteroids regulate organ boundary formation in the shoot apical meristem of Arabidopsis. Proceedings of the National Academy of Sciences, USA 109: 21152–21157. 10.1073/pnas.1210799110 PMC3529081 23213257

[nph71320-bib-0011] Gruszka D . 2020. Exploring the brassinosteroid signaling in monocots reveals novel components of the pathway and implications for plant breeding. International Journal of Molecular Sciences 21: 354. 31948086 10.3390/ijms21010354 PMC6982108

[nph71320-bib-0012] He JX , Gendron JM , Sun Y , Gampala SS , Gendron N , Sun CQ , Wang ZY . 2005. BZR1 is a transcriptional repressor with dual roles in brassinosteroid homeostasis and growth responses. Science 307: 1634–1638. 15681342 10.1126/science.1107580 PMC2925132

[nph71320-bib-0013] Hirano K , Yoshida H , Aya K , Kawamura M , Hayashi M , Hobo T , Sato‐Izawa K , Kitano H , Ueguchi‐Tanaka M , Matsuoka M . 2017. SMALL ORGAN SIZE 1 and SMALL ORGAN SIZE 2/DWARF AND LOW‐TILLERING form a complex to integrate auxin and brassinosteroid signaling in rice. Molecular Plant 10: 590–604. 28069545 10.1016/j.molp.2016.12.013

[nph71320-bib-0014] Hou J , Zheng X , Ren R , Shi Q , Xiao H , Chen Z , Yue M , Wu Y , Hou H , Li L . 2022. The histone deacetylase 1/GSK3/SHAGGY‐like kinase 2/BRASSINAZOLE‐RESISTANT 1 module controls lateral root formation in rice. Plant Physiology 189: 858–873. 35078247 10.1093/plphys/kiac015 PMC9157092

[nph71320-bib-0015] Hu D , Wei L , Liao W . 2021. Brassinosteroids in plants: crosstalk with small‐molecule compounds. Biomolecules 11: 1800. 34944444 10.3390/biom11121800 PMC8698649

[nph71320-bib-0016] Jiao Z , Yin L , Zhang Q , Xu W , Jia Y , Xia K , Zhang M . 2022. The putative obtusifoliol 14α‐demethylase OsCYP51H3 affects multiple aspects of rice growth and development. Physiologia Plantarum 174: e13764. 35975452 10.1111/ppl.13764

[nph71320-bib-0017] Jung C , Müller AE . 2009. Flowering time control and applications in plant breeding. Trends in Plant Science 14: 563–573. 19716745 10.1016/j.tplants.2009.07.005

[nph71320-bib-0018] Kim TW , Guan S , Burlingame AL , Wang ZY . 2011. The CDG1 kinase mediates brassinosteroid signal transduction from BRI1 receptor kinase to BSU1 phosphatase and GSK3‐like kinase BIN2. Molecular Cell 43: 561–571. 21855796 10.1016/j.molcel.2011.05.037 PMC3206214

[nph71320-bib-0019] Kim TW , Guan S , Sun Y , Deng Z , Tang W , Shang JX , Sun Y , Burlingame AL , Wang ZY . 2009. Brassinosteroid signal transduction from cell‐surface receptor kinases to nuclear transcription factors. Nature Cell Biology 11: 1254–1260. 19734888 10.1038/ncb1970 PMC2910619

[nph71320-bib-0020] Lee JH , Cho YS , Yoon HS , Suh MC , Moon J , Lee I , Weigel D , Yun CH , Kim JK . 2005. Conservation and divergence of *FCA* function between Arabidopsis and rice. Plant Molecular Biology 58: 823–838. 16240176 10.1007/s11103-005-8105-8

[nph71320-bib-0021] Li D , Wang L , Wang M , Xu YY , Luo W , Liu YJ , Xu ZH , Li J , Chong K . 2009. Engineering *OsBAK1* gene as a molecular tool to improve rice architecture for high yield. Plant Biotechnology Journal 7: 791–806. 19754838 10.1111/j.1467-7652.2009.00444.x

[nph71320-bib-0022] Li J , Wen J , Lease KA , Doke JT , Tax FE , Walker JC . 2002. BAK1, an Arabidopsis LRR receptor‐like protein kinase, interacts with BRI1 and modulates brassinosteroid signaling. Cell 110: 213–222. 12150929 10.1016/s0092-8674(02)00812-7

[nph71320-bib-0023] Liu X , Guo X , Li T , Wang X , Guan Y , Wang D , Wang Y , Ji X , Gao Q , Ji J . 2025. OsGSK1 interacts with OsbZIP72 to regulate salt response in rice. The Plant Journal 121: e70112. 40121668 10.1111/tpj.70112

[nph71320-bib-0024] Manghwar H , Hussain A , Ali Q , Liu F . 2022. Brassinosteroids (BRs) role in plant development and coping with different stresses. International Journal of Molecular Sciences 23: 1012. 35162936 10.3390/ijms23031012 PMC8835148

[nph71320-bib-0025] Meng F , Zheng X , Wang J , Qiu T , Yang Q , Fang K , Bhadauria V , Peng YL , Zhao W . 2024. The GRAS protein OsDLA involves in brassinosteroid signalling and positively regulates blast resistance by forming a module with GSK2 and OsWRKY53 in rice. Plant Biotechnology Journal 22: 363–378. 37794842 10.1111/pbi.14190 PMC10826986

[nph71320-bib-0026] Nam KH , Li J . 2002. BRI1/BAK1, a receptor kinase pair mediating brassinosteroid signaling. Cell 110: 203–212. 12150928 10.1016/s0092-8674(02)00814-0

[nph71320-bib-0027] Niu M , Wang H , Yin W , Meng W , Xiao Y , Liu D , Zhang X , Dong N , Liu J , Yang Y *et al*. 2022. Rice DWARF AND LOW‐TILLERING and the homeodomain protein OSH15 interact to regulate internode elongation via orchestrating brassinosteroid signaling and metabolism. Plant Cell 34: 3754–3772. 35789396 10.1093/plcell/koac196 PMC9516196

[nph71320-bib-0028] Qi PL , Zhou HR , Zhao QQ , Feng C , Ning YQ , Su YN , Cai XW , Yuan DY , Zhang ZC , Su XM *et al*. 2022. Characterization of an autonomous pathway complex that promotes flowering in Arabidopsis. Nucleic Acids Research 50: 7380–7395. 35766439 10.1093/nar/gkac551 PMC9303297

[nph71320-bib-0029] Qiao S , Sun S , Wang L , Wu Z , Li C , Li X , Wang T , Leng L , Tian W , Lu T *et al*. 2017. The RLA1/SMOS1 transcription factor functions with OsBZR1 to regulate brassinosteroid signaling and rice architecture. Plant Cell 29: 292–309. 28100707 10.1105/tpc.16.00611 PMC5354187

[nph71320-bib-0030] Ryu H , Cho H , Kim K , Hwang I . 2010. Phosphorylation dependent nucleocytoplasmic shuttling of BES1 is a key regulatory event in brassinosteroid signaling. Molecules and Cells 29: 283–290. 20387034 10.1007/s10059-010-0035-x

[nph71320-bib-0031] Shen Y , Yang G , Miao X , Shi Z . 2023. OsmiR159 modulate BPH resistance through regulating G‐protein γ subunit *GS3* gene in rice. Rice 16: 30. 37402009 10.1186/s12284-023-00646-z PMC10319700

[nph71320-bib-0032] Simpson GG , Dijkwel PP , Quesada V , Henderson I , Dean C . 2003. FY is an RNA 3′ end‐processing factor that interacts with FCA to control the Arabidopsis floral transition. Cell 113: 777–787. 12809608 10.1016/s0092-8674(03)00425-2

[nph71320-bib-0033] Song YH , Shim JS , Kinmonth‐Schultz HA , Imaizumi T . 2015. Photoperiodic flowering: time measurement mechanisms in leaves. Annual Review of Plant Biology 66: 441–464. 10.1146/annurev-arplant-043014-115555 PMC4414745 25534513

[nph71320-bib-0034] Sun D , Wu R , Zheng J , Li P , Yu L . 2018. Polyubiquitin chain‐induced p62 phase separation drives autophagic cargo segregation. Cell Research 28: 405–415. 29507397 10.1038/s41422-018-0017-7 PMC5939046

[nph71320-bib-0035] Tanaka K , Murata K , Yamazaki M , Onosato K , Miyao A , Hirochika H . 2003. Three distinct rice cellulose synthase catalytic subunit genes required for cellulose synthesis in the secondary wall. Plant Physiology 133: 73–83. 12970476 10.1104/pp.103.022442 PMC196581

[nph71320-bib-0036] Tang W , Kim TW , Oses‐Prieto JA , Sun Y , Deng Z , Zhu S , Wang R , Burlingame AL , Wang ZY . 2008. BSKs mediate signal transduction from the receptor kinase BRI1 in Arabidopsis. Science 321: 557–560. 18653891 10.1126/science.1156973 PMC2730546

[nph71320-bib-0037] Tian X , He M , Mei E , Zhang B , Tang J , Xu M , Liu J , Li X , Wang Z , Tang W *et al*. 2021. WRKY53 integrates classic brassinosteroid signaling and the mitogen‐activated protein kinase pathway to regulate rice architecture and seed size. Plant Cell 33: 2753–2775. 34003966 10.1093/plcell/koab137 PMC8408444

[nph71320-bib-0038] Tong H , Chu C . 2018. Functional specificities of brassinosteroid and potential utilization for crop improvement. Trends in Plant Science 23: 1016–1028. 30220494 10.1016/j.tplants.2018.08.007

[nph71320-bib-0039] Tong H , Jin Y , Liu W , Li F , Fang J , Yin Y , Qian Q , Zhu L , Chu C . 2009. DWARF AND LOW‐TILLERING, a new member of the GRAS family, plays positive roles in brassinosteroid signaling in rice. The Plant Journal 58: 803–816. 19220793 10.1111/j.1365-313X.2009.03825.x

[nph71320-bib-0040] Tong H , Liu L , Jin Y , Du L , Yin Y , Qian Q , Zhu L , Chu C . 2012. DWARF AND LOW‐TILLERING acts as a direct downstream target of a GSK3/SHAGGY‐like kinase to mediate brassinosteroid responses in rice. Plant Cell 24: 2562–2577. 22685166 10.1105/tpc.112.097394 PMC3406904

[nph71320-bib-0041] Wang H , Jiao X , Kong X , Liu Y , Chen X , Fang R , Yan Y . 2020. The histone deacetylase HDA703 interacts with OsBZR1 to regulate rice brassinosteroid signaling, growth and heading date through repression of *Ghd7* expression. The Plant Journal 104: 447–459. 33617099 10.1111/tpj.14936

[nph71320-bib-0042] Wang H , Ye T , Guo Z , Yao Y , Tu H , Wang P , Zhang Y , Wang Y , Li X , Li B *et al*. 2024. A double‐stranded RNA binding protein enhances drought resistance via protein phase separation in rice. Nature Communications 15: 2514. 10.1038/s41467-024-46754-2 PMC10957929 38514621

[nph71320-bib-0043] Wang W , Wang C , Wang Y , Ma J , Wang T , Tao Z , Liu P , Li S , Hu Y , Gu A *et al*. 2023. The P‐body component DECAPPING5 and the floral repressor SISTER OF FCA regulate FLOWERING LOCUS C transcription in Arabidopsis. Plant Cell 35: 3303–3324. 37220754 10.1093/plcell/koad151 PMC10473201

[nph71320-bib-0044] Wang X , Chory J . 2006. Brassinosteroids regulate dissociation of BKI1, a negative regulator of BRI1 signaling, from the plasma membrane. Science 313: 1118–1122. 16857903 10.1126/science.1127593

[nph71320-bib-0045] Xing HL , Dong L , Wang ZP , Zhang HY , Han CY , Liu B , Wang XC , Chen QJ . 2014. A CRISPR/Cas9 toolkit for multiplex genome editing in plants. BMC Plant Biology 14: 327. 25432517 10.1186/s12870-014-0327-y PMC4262988

[nph71320-bib-0046] Xu C , Wu Z , Duan HC , Fang X , Jia G , Dean C . 2021. R‐loop resolution promotes co‐transcriptional chromatin silencing. Nature Communications 12: 1790. 10.1038/s41467-021-22083-6 PMC7979926 33741984

[nph71320-bib-0047] Yamamuro C , Ihara Y , Wu X , Noguchi T , Fujioka S , Takatsuto S , Ashikari M , Kitano H , Matsuoka M . 2000. Loss of function of a rice brassinosteroid insensitive1 homolog prevents internode elongation and bending of the lamina joint. Plant Cell 12: 1591–1606. 11006334 10.1105/tpc.12.9.1591 PMC149072

[nph71320-bib-0048] Yin W , Dong N , Li X , Yang Y , Lu Z , Zhou W , Qian Q , Chu C , Tong H . 2025. Understanding brassinosteroid‐centric phytohormone interactions for crop improvement. Journal of Integrative Plant Biology 67: 563–581. 39927447 10.1111/jipb.13849

[nph71320-bib-0049] Yin Y , Vafeados D , Tao Y , Yoshida S , Asami T , Chory J . 2005. A new class of transcription factors mediates brassinosteroid‐regulated gene expression in Arabidopsis. Cell 120: 249–259. 15680330 10.1016/j.cell.2004.11.044

[nph71320-bib-0050] Yoshida S , Forno D , Cock J . 1971. Laboratory manual for physiological studies of rice. Manila: The International Rice Research Institute. 83 p.

[nph71320-bib-0051] Yu X , Li L , Zola J , Aluru M , Ye H , Foudree A , Guo H , Anderson S , Aluru S , Liu P *et al*. 2011. A brassinosteroid transcriptional network revealed by genome‐wide identification of BESI target genes in *Arabidopsis thaliana* . The Plant Journal 65: 634–646. 21214652 10.1111/j.1365-313X.2010.04449.x

[nph71320-bib-0052] Zhang B , Wang X , Zhao Z , Wang R , Huang X , Zhu Y , Yuan L , Wang Y , Xu X , Burlingame AL *et al*. 2016. OsBRI1 activates BR signaling by preventing binding between the TPR and kinase domains of OsBSK3 via phosphorylation. Plant Physiology 170: 1149–1161. 26697897 10.1104/pp.15.01668 PMC4734578

[nph71320-bib-0053] Zhang C , Xu Y , Guo S , Zhu J , Huan Q , Liu H , Wang L , Luo G , Wang X , Chong K . 2012. Dynamics of brassinosteroid response modulated by negative regulator LIC in rice. PLoS Genetics 8: e1002686. 22570626 10.1371/journal.pgen.1002686 PMC3343102

[nph71320-bib-0054] Zhang Z , Tan J , Shi Z , Xie Q , Xing Y , Liu C , Chen Q , Zhu H , Wang J , Zhang J *et al*. 2016. *Albino leaf1* that encodes the sole octotricopeptide repeat protein is responsible for chloroplast development. Plant Physiology 171: 1182–1191. 27208287 10.1104/pp.16.00325 PMC4902615

