# Supplementary material for: The GSK3/SHAGGY‐like OsGSK3 phosphorylates and inhibits phase separation of OsFCA at Ser‐43 and Ser‐45 to regulate brassinosteroid signaling and rice architecture
Source: New Phytol. 2026 Jun 4;251(4):1909–29. doi: 10.1111/nph.71320 (PMC13373847; doi:10.1111/nph.71320)
Supplement: Supplementary file 1 — Fig. S1 Cluster heatmap of proteins showing consistent upregulation or downregulation trends across treatments. Fig. S2 Y2H analysis of interactions between OsGSK3 and candidates from the phosphoproteome. Fig. S3 Phenotypes of the m‐Osfca mutant. Fig. S4 Identification of Osfca mutants. Fig. S5 Identification of Osgsk3 and Osgsk3 Osfca mutants. Fig. S6 OsFCA contributes to OsGSK3‐mediated BR signal transduction. Fig. S7 Cluster heatmap of all DEGs in ZH11, Osfca, and Osgsk3 Osfca. Fig. S8 OsFCA transcription exhibits a circadian rhythmic. Fig. S9 Identification of OsFCA phosphorylation sites. Table S1 Primers used in this study. Table S2 Protein groups identified. Table S3 Unique peptides identified. Table S4 Phosphopeptides identified. Table S5 Phosphorylation sites identified. Table S6 Quantifiable protein entries. Table S7 Representative phosphorylation sites. Table S8 Proteins associated with OsGSK3 and BR signaling. Table S9 Proteins forming the protein–protein interaction (PPI) network with OsGSK3 as the central node. Please note: Wiley is not responsible for the content or functionality of any Supporting Information supplied by the authors. Any queries (other than missing material) should be directed to the New Phytologist Central Office. [file NPH-251-1909-s001.pdf]

New Phytologist Supporting Information

Article title: The GSK3/SHAGGY-Like OsGSK3 Phosphorylates and Inhibits Phase Separation of OsFCA at Ser-43 and Ser-45 to Regulate Brassinosteroid Signaling and Rice Architecture

Authors: Jiaqi Zhang, Fan Wang, Sijia Zhang, Qian Yu, Qimiao Dong, Jianbo Li, Xianglei Wei, Huaying Du, Ye Shen, Rong Mu, Yanxiao Jia, Jinping Cheng, Hongsheng Zhang, Ji Huang and Xiuying Gao

Article acceptance date: 17 May 2026

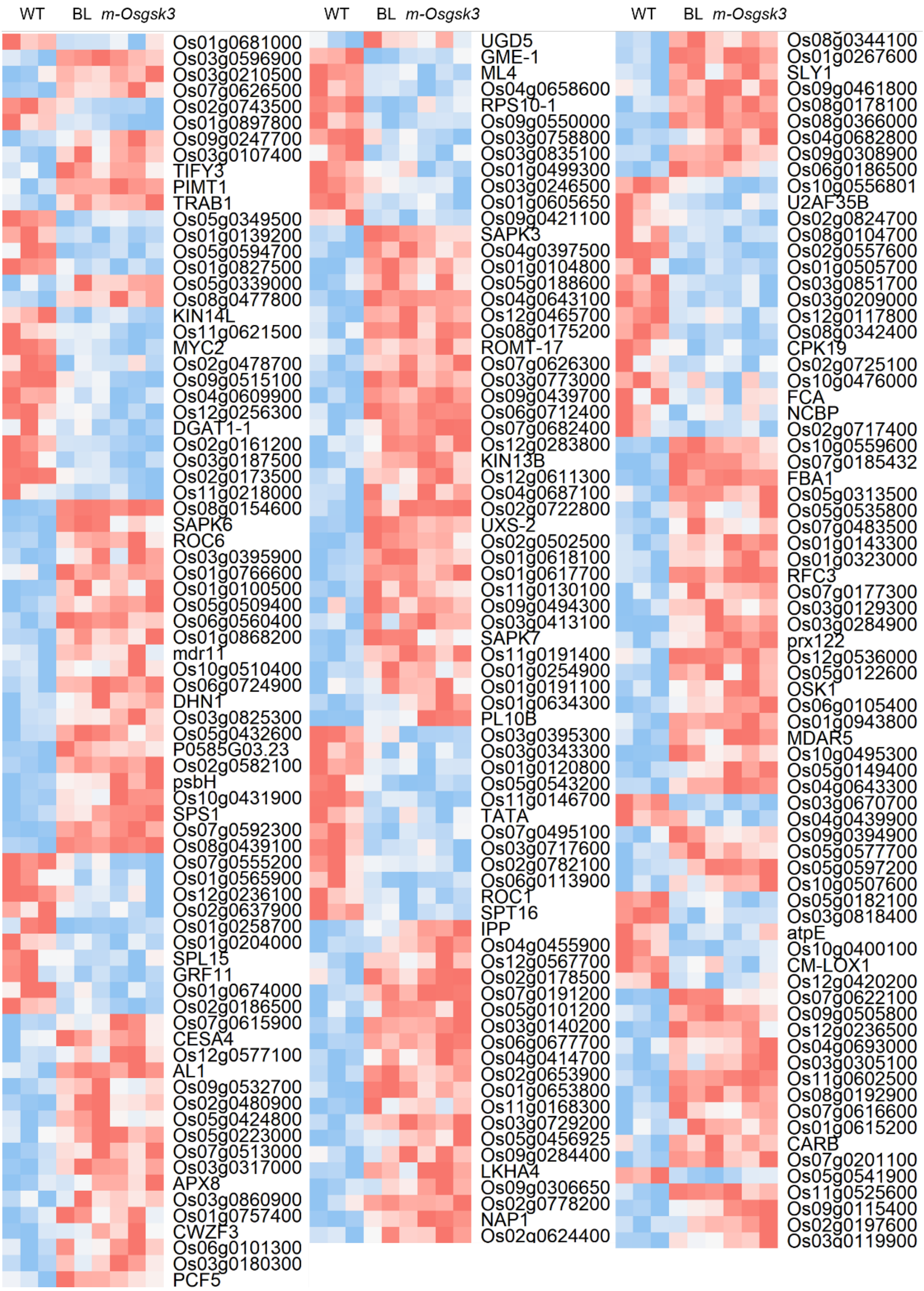

Fig. S1 Cluster heatmap of proteins showing consistent upregulation or downregulation trends across treatments.

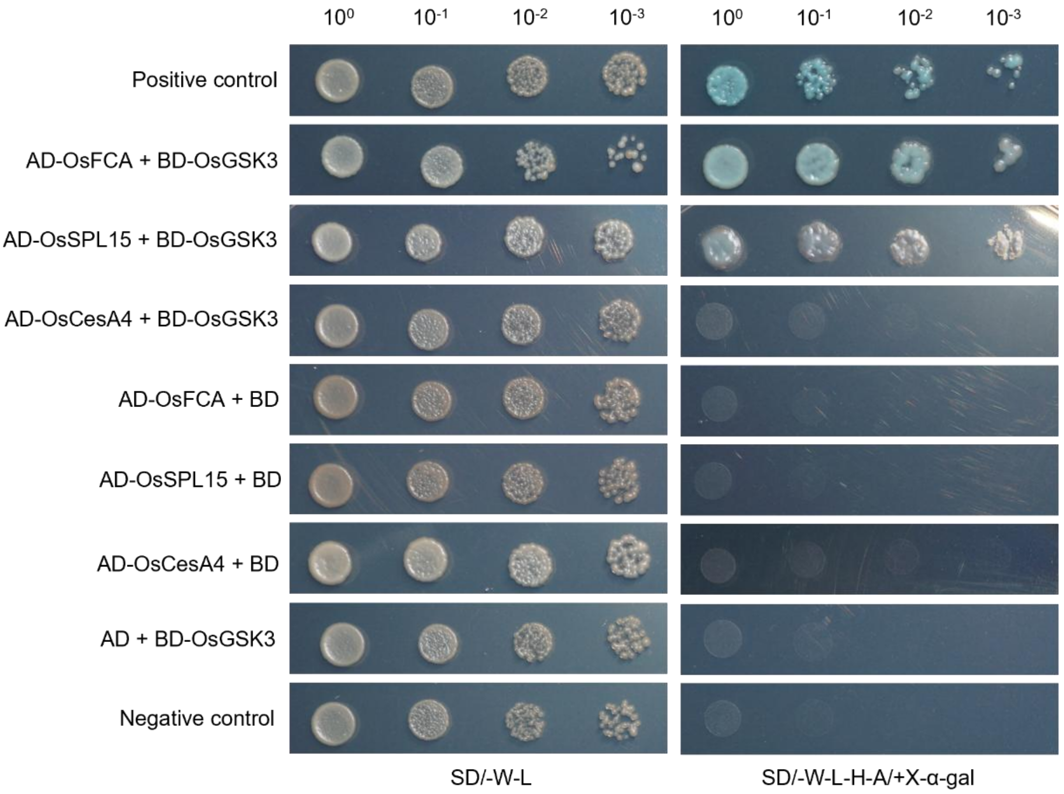

Fig. S2 Y2H analysis of interactions between OsGSK3 and candidates from the phosphoproteome. Yeast two-hybrid (Y2H) assay showing that OsFCA and OsSPL15 interacts with OsGSK3 in yeast cells. Yeast cultures carrying the indicated combinations of plasmids were serially diluted with ddH_2O (10^0, 10^{-1}, 10^{-2}, 10^{-3}) and spotted onto synthetic defined (SD) medium lacking tryptophan and leucine (SD/−W−L) and SD medium lacking tryptophan, leucine, histidine, and adenine, and containing X-α-gal (SD/−W−L−H−A/+X-α-gal).

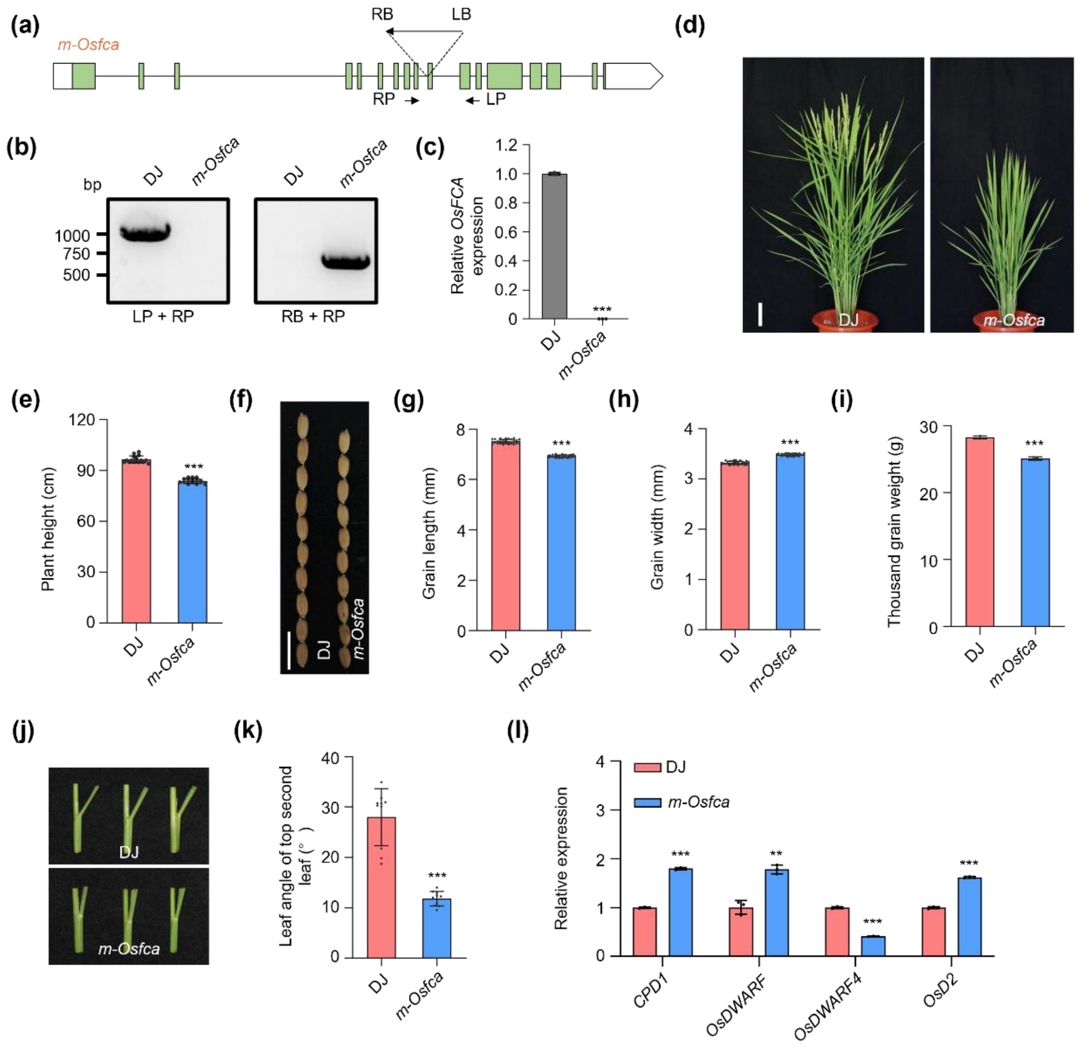

Fig. S3 Phenotypes of the *m-Osfca* mutant. (a) Diagram of the *OsFCA* locus showing the position of the T-DNA insertion in m-*Osfca* mutant. Green boxes indicate exons, white boxes indicate untranslated regions (UTRs), and lines indicate introns. LP, left gene-specific primer; RP, right gene-specific primer; LB, T-DNA left border; RB, T-DNA right border. (b) PCR verification of the T-DNA insertion site in *m-Osfca*. (c) RT-qPCR analysis of relative *OsFCA* expression levels in Dongjin (DJ) and the m-*Osfca* mutant. Data are means ± SD (n = 3). (d) Representative profile photographs of DJ and *m-Osfca* plants at the mature stage. Scale bar, 10 cm. (e) Statistical data on plant height of DJ and *m-Osfca* plants. Data are means ± SD (n = 15). (f) Grain size of DJ and *m-Osfca* plants. Scale bar, 1 cm. (g, h) Statistical data on grain length (g) and grain width (h) of DJ and *m-Osfca* plants. Data are means ± SD (n = 30). (i) Statistical data on thousand-grain weight of DJ and *m-Osfca* plants. Data are means ± SD (n = 3). (j) Representative photograph showing the angle of the second top leaf of DJ and *m-Osfca* plants at the grain-filling stage. (k) Statistical data on angle of the second top leaf at the grain-filling stage. Data are means ± SD (n = 10). (l) RT-qPCR analysis of the expression levels of BR biosynthesis-related genes in DJ and *m-Osfca*. Data are means ± SD (n = 3). In (e, g-i, k, l), asterisks indicate significant differences compared with the wild type, as determined by a Student's t-test; **, P < 0.01; ***, P < 0.001.

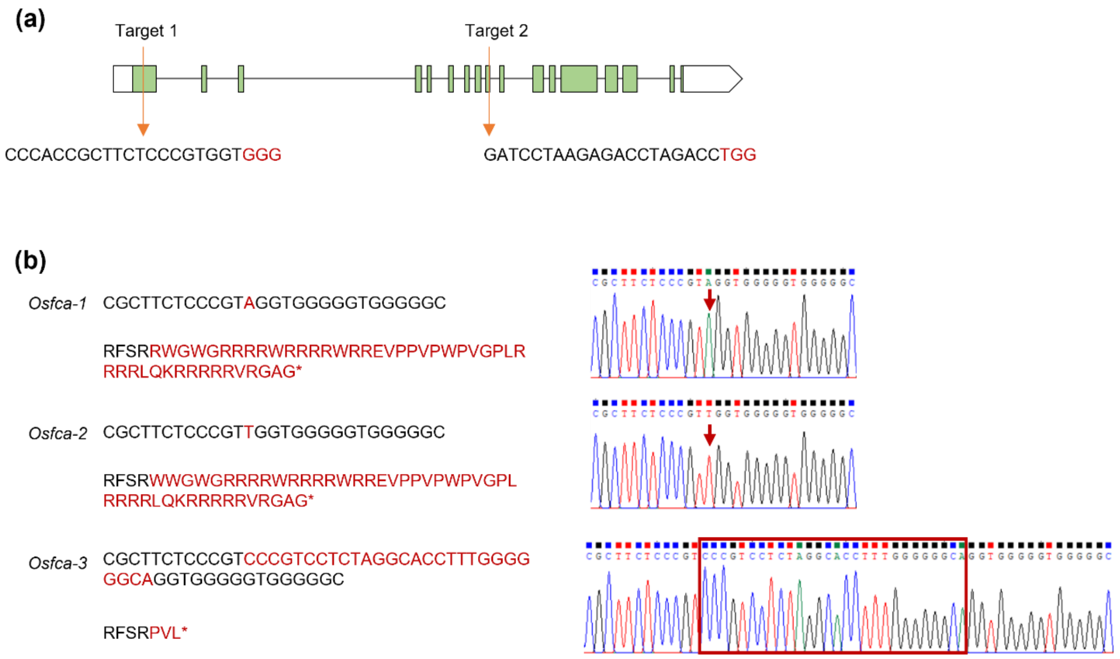

Fig. S4 Identification of *Osfca* mutants. (a) Gene-editing targets for *Osfca* materials. Green boxes indicate exons, white boxes indicate UTRs, and lines between boxes are introns. Target 1 and Target 2 were designed on the website. The red-labeled bases in the target sequence are PAM. (b) Sequencing peak diagram of *Osfca* materials and amino acid translation changes caused by mutations. The positions of red arrows and red boxes in the peak diagram represent the sites where the mutations occurred.

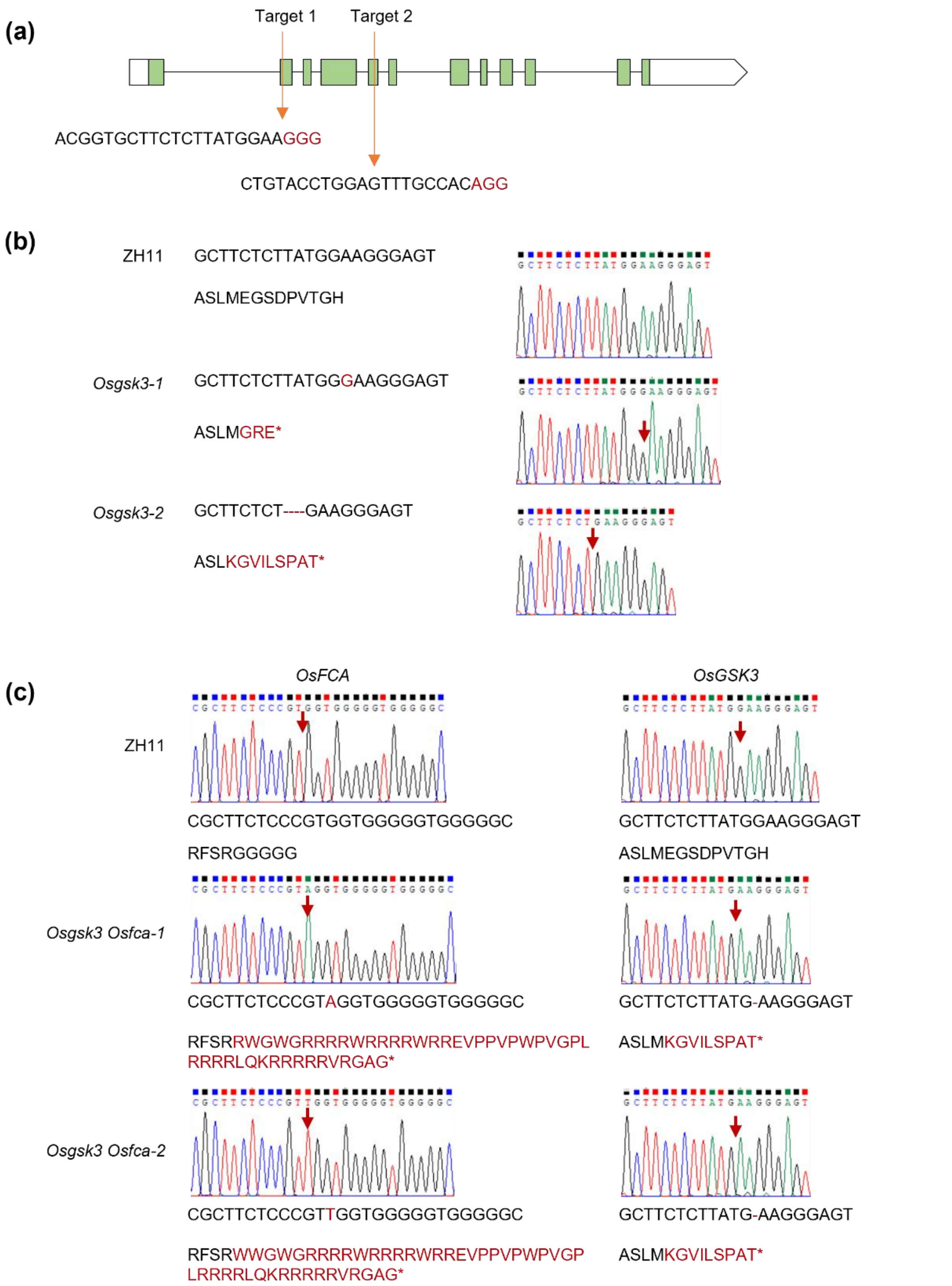

Fig. S5 Identification of *Osgsk3* and *Osgsk3 Osfca* mutants. (a) Gene-editing targets for *Osgsk3* materials. Green boxes indicate exons, white boxes indicate UTRs, and lines between boxes are introns. Target 1 and Target 2 were designed on the website. The red-labeled bases in the target sequence are PAM. (b, c) Sequencing peak diagram of *Osgsk3*(b) and *Osgsk3 Osfca* (c) materials and amino acid translation changes caused by mutations. The red letters marked in the nucleotide sequence are insertion bases, and the red horizontal lines represent base deletions. The positions of red arrows in the peak diagram are the sites where the mutations occurred.

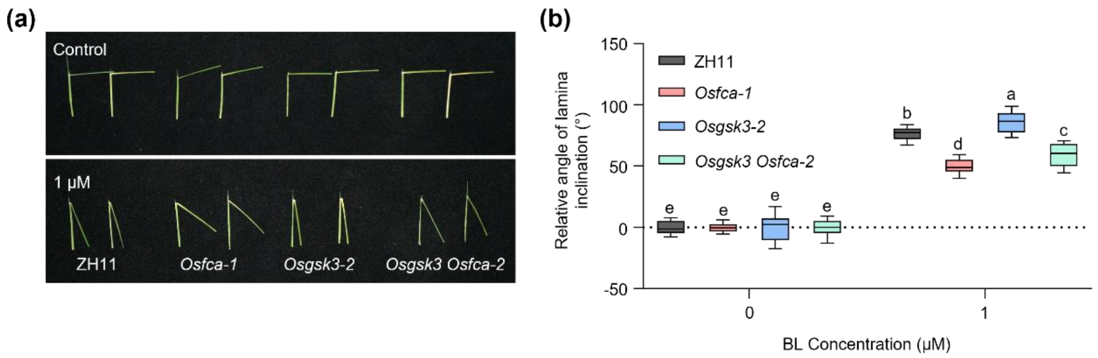

Fig. S6 OsFCA contributes to OsGSK3-mediated BR signal transduction. (a) Lamina inclination assay in ZH11, *Osfca-1*, *Osgsk3-2*, and *Osgsk3 Osfca-2* plants in response to treatment with 1 μM 24-eBL. (b) Quantification of lamina inclination in ZH11, *Osfca-1*, *Osgsk3-2*, and *Osgsk3 Osfca-2* plants. Data are means ± SD (n = 30). The horizontal line within each box indicates the median, the box bounds the interquartile range, and the whiskers extend to the minimum and maximum values. Different lowercase letters denote a significant difference between means, as determined by Duncan's multiple range tests ($P < 0.05$).

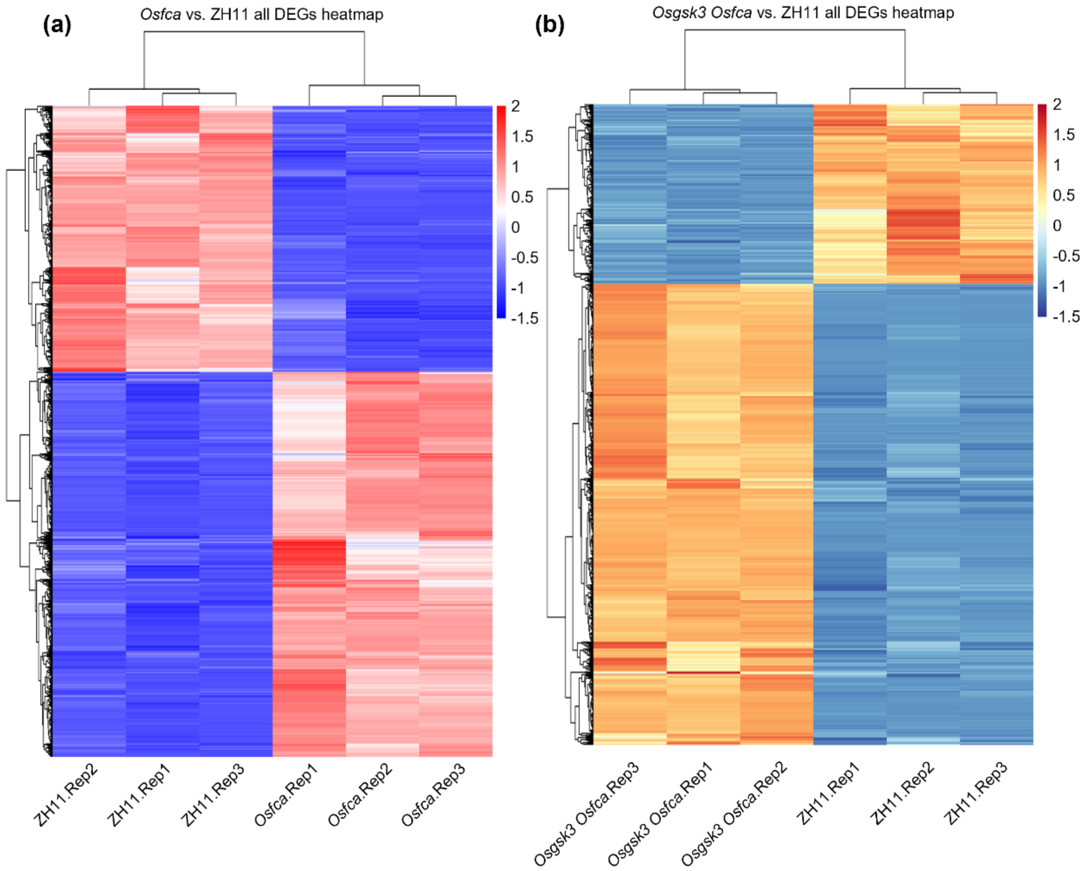

Fig. S7 Cluster heatmap of all DEGs in ZH11, *Osfca*, and *Osgsk3 Osfca*. (a) Cluster heatmap of all DEGs in young panicles of *Osfca* and ZH11 with three biological replicates per genotype. Scale bar shows fold changes, values are normalized by z-score scheme, red and blue colors indicate up- and down-regulated, respectively. (b) Cluster heatmap of all DEGs in young panicles of *Osgsk3 Osfca* and ZH11 with three biological replicates per genotype. Scale bar shows fold changes, values are

normalized by z-score scheme, orange and cerulean colors indicate up- and down-regulated, respectively.

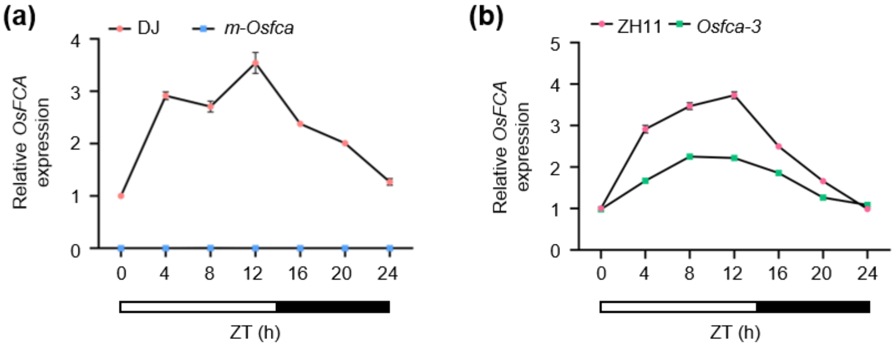

Fig. S8 *OsFCA* transcription exhibits a circadian rhythmic. (a) RT-qPCR analysis of *OsFCA* expression in DJ and *m-Osfca*. Data are means ± SD (n = 3). ZT, Zeitgeber time. (b) RT-qPCR analysis of *OsFCA* expression in ZH11 and *Osfca-3*. Data are means ± SD (n = 3). ZT, Zeitgeber time.

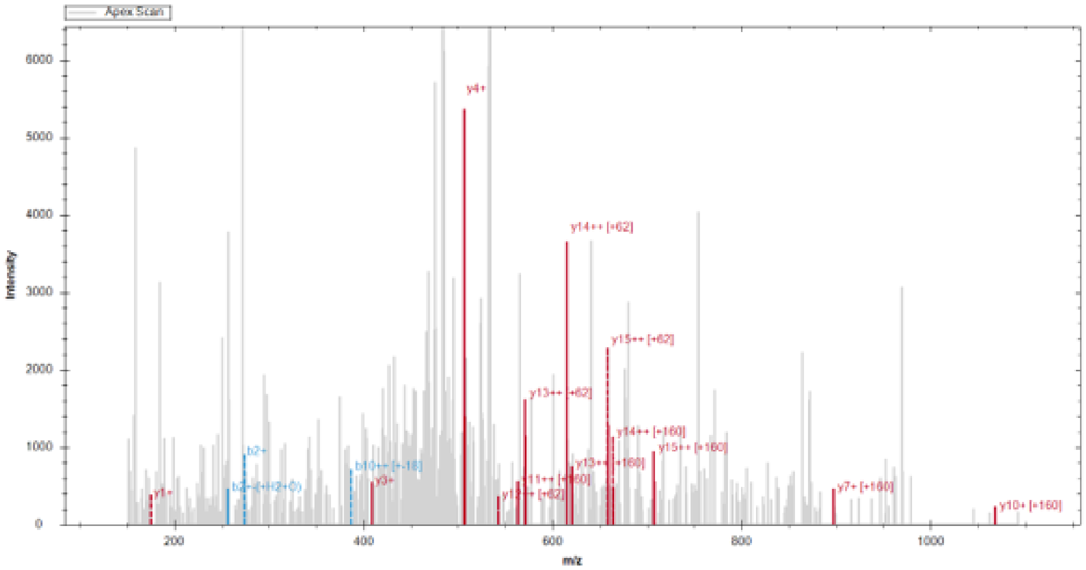

Fig. S9 Identification of OsFCA phosphorylation sites.
